# In vivo replication of a fully functional nerve net in a clonally reproducing animal

**DOI:** 10.1038/s42003-026-11184-x

**Published:** 2026-10-22

**Authors:** Ehsan Sakib, Lisa-Marie Hofacker, Christopher Noack, Finn Ströbel, Christoph Giez, Jörg Wittlieb, Alexander Klimovich, Thomas C. G. Bosch

**Affiliations:** 1 Zoological Institute, Christian-Albrechts University of Kiel, Kiel, Germany; 2 Present Address: Max-Planck-Institute for Evolutionary Biology, Plön, Germany; 3 Present Address: Department of Neurosciences and Developmental Biology, University of Vienna, Vienna, Austria; 4 Present Address: Neural Circuits and Evolution Laboratory, Francis Crick Institute, London, UK

**Keywords:** Neural circuits, Neuronal development

## Abstract

Nerve nets are considered the evolutionarily oldest form of nervous system. How such networks develop and how complex nervous systems become fully functional is largely unknown. In a phylogenetically ancient animal, the freshwater polyp *Hydra*, we demonstrate how a nerve net consisting of distinct neuronal populations is precisely replicated over the course of asexual proliferation by budding. During this process, precursor cells first accumulate at the site of the emerging neural network, followed by a stepwise formation of functional neural circuits composed of distinct neuronal populations. These circuits become arranged according to an accurately controlled spatiotemporal pattern and take control of behaviors independently of the existing parental network. Since similar processes also govern the formation of more complex nervous systems in other invertebrates and vertebrates, we suggest that the basic requirements for a neuronal network to replicate were already in place at the beginning of the evolution of the nervous system.

## Introduction

Nerve nets are considered the evolutionarily oldest organization of the nervous system, the major organ system responsible for processing information and coordinating animal behavior^1,2^. How diverse and complex nervous systems evolved from the simple nerve nets over the course of 600 million years remains one of the unresolved questions in evolutionary and comparative neurobiology. How do neurons form functional units to orchestrate simple behaviors, and what are the minimal requirements for a functional neuronal circuit? Addressing these questions requires a model system that is both experimentally traceable and evolutionarily informative.

**Fig. 1:**
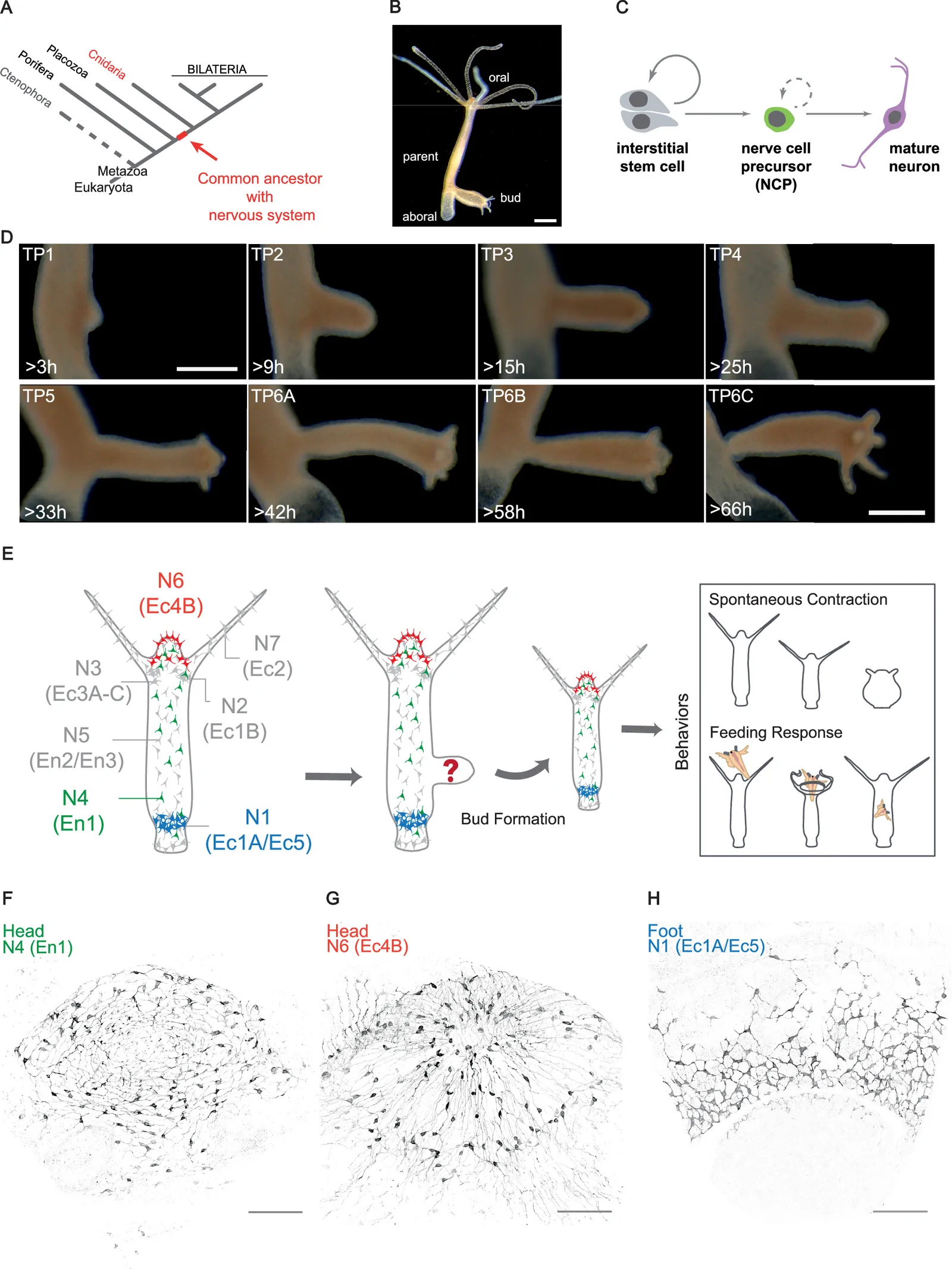
The *Hydra* budding paradigm: a model for nerve net replication in a clonally reproducing animal. **A** Phylogenetic position of *Hydra* within the phylum Cnidaria. The origin of the nervous system has preceded the radiation of Cnidaria and Bilateria. **B** Image of a budding *Hydra vulgaris* polyp with an attached bud. Scale bar: 500 µm. **C** Schematic overview of neurogenesis in *Hydra*: mature neurons develop from intensively self-renewing (*solid arrow*) interstitial stem cells through a transient state of a migratory nerve cell precursor (NCP) with a limited proliferative capacity (*dashed arrow*). **D** Overview of distinct morphological stages during the development of a *Hydra* bud, which progresses over approximately 66 h. **E** Schematic summary of all neuronal populations in *Hydra vulgaris* identified via single-cell RNA sequencing, demonstrating correspondence of nomenclature used in different studies and highlighting the three populations investigated in this study—N1, N4, and N6. The enigmatic question of how these populations are formed in a developing bud and how they contribute to the onset of specific behaviors during the budding process is the focus of this study. **F** Immunohistochemical detection of the endodermal neuronal population N4, image from the hypostome region of an adult *Hydra*. Scale bar: 50 μm. **G** Immunohistochemical staining of the ectodermal population N6 located in the hypostome around the mouth opening of an adult *Hydra*. Scale bar: 50 μm. **H** Immunohistochemical detection of the ectodermal population N1 located around the peduncle of an adult *Hydra*. Scale bar: 50 μm.

Cnidarians, members of the phylum that diverged from their sister group Bilateria (Fig. 1A) over 650 million years ago, occupy a key phylogenetic position for investigating the evolution of nervous systems^3,4^. Molecular studies provided solid evidence that the first neurons and nervous systems appeared in the common cnidarian-bilaterian ancestor^5^ and cnidarian nervous systems operate using remarkably similar molecular machinery as the bilaterian ones^6^. Hence, cnidarian nerve nets have great potential for revealing the fundamental principles of nervous systems and the evolution of their complexity^6–8^.

Across animal phyla, neurogenesis, i.e., the process by which new specialized neurons are generated from neural stem cells, is fundamental for nerve net development and growth. In contrast to most model organisms^9^, neurogenesis and nerve net formation in the freshwater cnidarian *Hydra* (Fig. 1B) are not restricted to embryogenic development but occur continuously in adult polyps (Fig. 1C). This allows lifelong homeostasis and renewal of the nerve net in a polyp^10–12^. Another unique feature of *Hydra* is the polyp’s asexual reproduction (Fig. 1B, D), where an outgrowth from the parent’s body, termed a bud, develops into a clonal copy of the parent polyp and separates to become an independent polyp within just 60–70 h^13^. During this development, both the ectoderm and the endoderm remain as adjacent epithelial layers, where the endoderm of the bud is a physical continuation of the parental polyp’s endoderm^14^. Throughout the bud’s development, a functional nerve net forms, which may ultimately enable the newly formed young polyp to display an entire behavioral repertoire characteristic of an adult polyp. Continuous neurogenesis likely contributes to the bud’s nerve net formation, but how this new, independent neuronal network develops in the bud and how it orchestrates behavior remains unknown.

In adult *Hydra* polyps, all neurons emerge from interstitial stem cells (ISCs, Fig. 1C), which are located ectodermally in the body column, self-renew, and give rise to committed nerve cell precursors (NCPs)^12,15^. These precursors migrate and undergo terminal mitosis prior to their differentiation into mature functional neurons in situ^13,16^. In adult polyps, 7 to 11 molecularly distinct populations of neurons reside in specific regions along the body column^17–21^. The NCPs contribute to the formation of all ectodermal neuronal populations—N1, N2, N3, N6, and N7—while also giving rise to transient precursors that migrate through the mesoglea into the endoderm and give rise to the endodermal neuronal populations– N4 and N5^18,19^. Two morphological types of nerve cells compose the nerve net in *Hydra*: sensory and ganglion neurons^22^. The ganglion neurons are characterized by the proximity of their somata and multiple projections (typically 3–5) to the mesoglea, between the muscular protrusions of the epithelial cells. The somata of sensory neurons are located closer to the surface of epithelial cells, reach the epithelial surface with their sensory cilia, and their projections (typically 1–2) are located between the muscular protrusions of the epithelial cells^23^.

Mapping the marker gene expression by in *situ* hybridization, localization of distinct proteins by immunohistochemistry, analysis of transgenic reporter lines, and transmission electron microscopy provided evidence that while several neuronal populations comprise cells of only one type (N1, N2—only ganglion neurons; N5, N7—only sensory neurons), three neuronal populations contain both sensory and ganglion nerve cells (N3, N4, and N6)^18,19,21,24,25^. Interestingly, this morphological difference is not reflected in the molecular identity of neurons. For instance, we demonstrated earlier that the population of endodermal neurons, N4, consists of both sensory and ganglion neurons, which are functionally distinct. In contrast to that, no heterogeneity within the N4 population has been detected at the transcriptome level^18,19^. This points to a remarkable functional diversity of *Hydra* neurons. These spatially restricted neuronal populations (Fig. 1E, F) jointly orchestrate a rich behavioral repertoire of *Hydra*, including spontaneous recurrent contractions and extension of the body, complex feeding behavior, and somersaulting locomotion, as well as tuning of *Hydra’s* internal states^19,20,24,26–31^. The use of transgenesis approaches, such as calcium imaging of neuronal activity and targeted neuronal ablation, combined with quantitative behavioral analysis, enables interrogation of the neuronal circuit architecture and function in *Hydra*^24,32^. These approaches revealed a surprisingly complex relationship between distinct neuronal populations and behaviors: one behavior, in most cases, is a product of the interaction of several different neuronal populations^24,26^. However, the question of how these individual populations emerge, become interconnected, and functionally integrate in a developing bud remains open.

Here, using transgenic lines, we trace nerve net formation during asexual reproduction of *Hydra*. We uncover that local accumulation of NCPs in the budding zone precedes the formation of neuronal populations. Sequential formation of N4 (En1) and N6 (Ec4B) networks results in a stepwise assembly of the neuronal circuits, enabling precise control of mouth opening behavior. During late bud development, the N1 (Ec1A/Ec5) population forms at the foot of the bud, ensuring the uniformity of spontaneous contractions that are vital for the polyp. Our observations unveil the critical role of increasing connectivity and activity synchronization in the maturation of the emerging nerve net. The ordered formation of neural circuits appears to be an ancient evolutionary mechanism that not only ensures the correct architectural build of nervous systems but also sets the stage for complex behaviors.

## Results

### Local accumulation of nerve precursor cells precedes the formation of specific neuronal populations

**Fig. 2:**
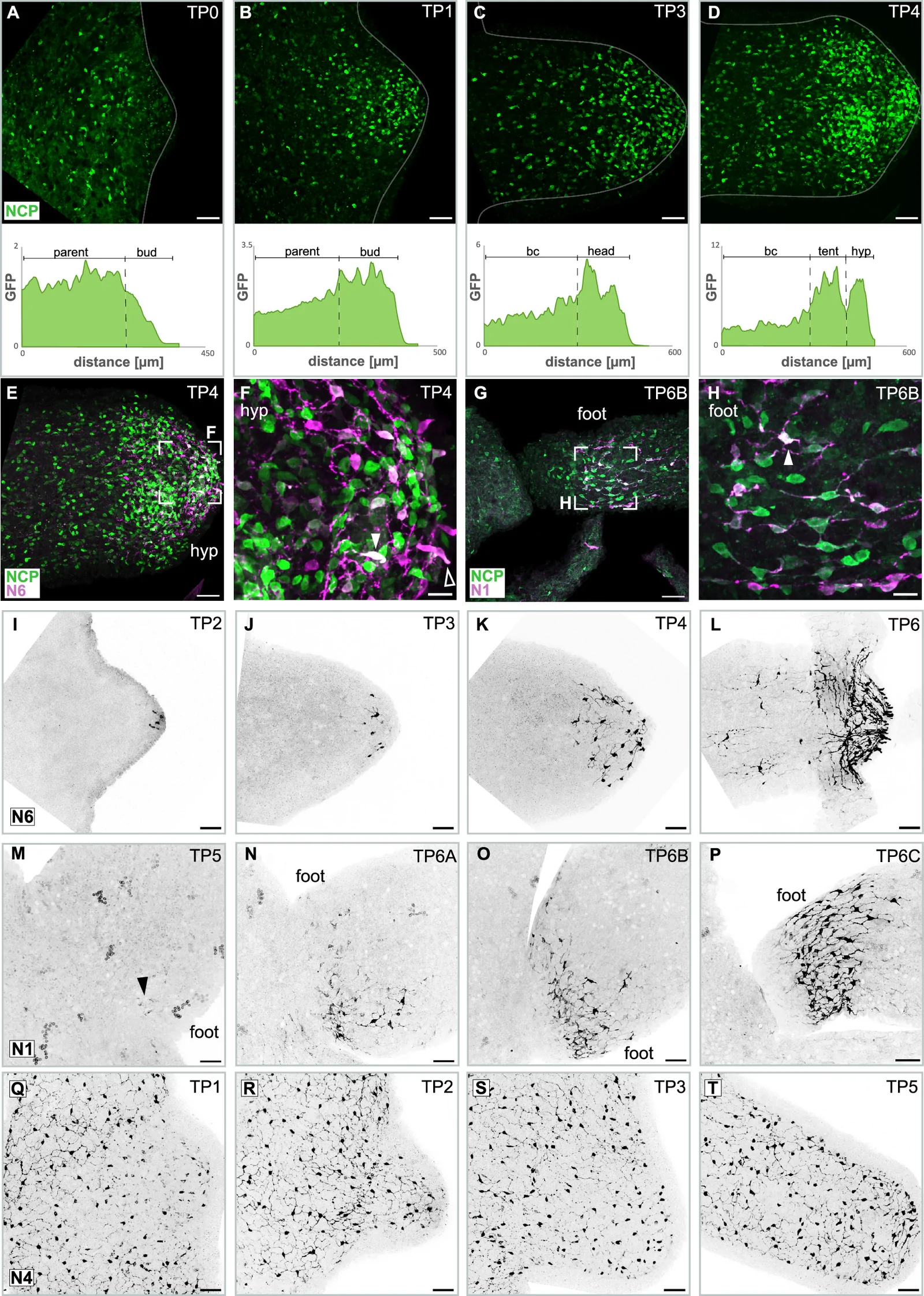
Pattern formation of NCPs precedes the formation of specific neuronal populations. **A**–**D** NCPs accumulate in the early bud evagination, form a dense cluster at the tip of a growing bud, and split into two populations at late developmental stages. Immunohistochemical detection of GFP-labeled NCPs in the budding zone of transgenic polyps and corresponding GFP signal density diagrams (arbitrary unit × 10^3^). Representative images and profiles of TP0-4 buds are shown, *n* = 3–5; parent—body column of the parental polyp; bud—emerging bud protrusion; bc—body column of the growing bud; head, hyp, tent—head, tentacles, and hypostome of the growing bud, correspondingly. See also Fig. S1. **E**–**H** NCPs gradually differentiate into mature neurons. Immunochemical detection of NCPs (GFP, *green*) and mature neurons (RFamide; *magenta*). The transition in expression of molecular markers at TP4 (GFP from NCPs, RFamide for N6 neurons) from green via white (overlap of green and magenta) to magenta indicates progressive differentiation of NCPs into mature neurons in a spatially and temporally controlled manner. In the distal tip, NCPs give rise to RFamide-positive N6 neurons (**E**, close-up on **F**). In the region proximal to the parental polyp, N1 RFamide neurons appear (**G**, close-up on **H**). Note the white cells (*arrowheads* on **F** and **H**) corresponding to GFP-positive and RFamide-positive cells transiting from GFP to RFamide expression during maturation. Also note N6 sensory neurons (white outlined *arrowhead* on **F**). **I**–**L** Emergence of the N6 network in a bud visualized in transgenic GFP-expressing polyps. Immunohistochemical detection of mature N6 neurons (GFP). Note the early appearance of the first N6 neurons (TP2 on **I**), gradual expansion and development of connectivity (**J**–**L**), and high density of N6 sensory neurons at the hypostome tip at the later stage (TP6 on **L**). **M**–**P** Emergence of the N1 network in a prospective foot region of a bud visualized with RFamide staining. Immunochemical detection of mature N1 neurons (RFamide) reveals the emergence and expansion of population N1 (TP5 on **M**) and its rapid expansion during late bud development (TP6A-C on **N**–**P**). **Q**–**T** Dynamics of the endodermal N4 population in the parental polyp and emerging bud visualized by immunochemical detection of mature N4 neurons (GFP) in transgenic polyps. Note the local disintegration of the N4 network in the parental polyp next to the bud protrusion (TP1 on **Q**) and high connectivity of the N4 network in the bud at later time points (TP2-5 on **R**–**T**). Images from representative polyps, scale bar 50 µm (**A**–**D**, **E**, **G**, **I**–**T**) or 15 µm (**F**, **H**).

All neurons in *Hydra* originate from ISCs through a transient migrating NCP state (Fig. 1C). To investigate the contribution of NCPs to the nerve net formation in a growing bud, we followed the NCPs in a novel transgenic *Hydra* line (myc3::GFP//act::DsRED; see “Transgenesis and constructs” in “Materials and methods”; Figs. S1–S4), where NCPs were labeled by expressing GFP under the control of the *myc3* promoter (*HVAEP_G003730*), highly active in the neuronal lineage^16,18^. In the parental polyps, NCPs are homogeneously distributed in the middle gastric region. However, simultaneously with the emergence of an epithelial evagination in the budding region (TP0, Figs. 2A, S1A and S2A, A′), the density of NCPs increases locally beneath the bud anlage, consistent with earlier observations^33^. As the outgrowth of the bud begins, the NCPs continue to accumulate in its most distal part (TP1, Figs. 2B, S1B and 2C, C′). With further growth (TP1-3), the highest density of NCPs is consistently observed at the distal tip of the bud, which is the prospective head (Figs. 2B, C, S1B–E, S2C–D′ and S3A, B). At the later time point (TP4), the local population of NCPs segregates into different populations, corresponding to prospective hypostome and tentacles (Figs. 2D, S1F, G and S3C). To gain first insights into the differentiation of these spatially allocated NCP populations, we simultaneously detected mature neurons labeled by DsRED expression in the emerging bud in the same transgenic polyps (myc3::GFP// act::DsRED; Figs. S1I–L and S4). Immunostaining against DsRED revealed that only scattered differentiated neurons are present in early buds (TP2-3; Fig. S1I, J), but an accumulation of mature neurons at the hypostome tip becomes conspicuous at TP4-5 (Fig. S1K, L). This suggests that the expansion of the NCP population at the emerging bud tip is accompanied by their local differentiation. To gain further insights into the identity of these emerging neurons, we performed immunostaining against the RFamide neuropeptide, which is expressed in multiple neuronal populations (N6, N7, and N1; Fig. 1E, G, H)^19,24^. In the emerging bud, RFamide-positive neurons appear in the hypostome tip, which, based on their spatial localization, is consistent with the position of the N6 population in adult polyps (Fig. 2E, F)^19,24^. A transition in the expression of molecular markers at TP4 enables the visualization of emerging N6 neurons (RFamide, magenta) that progressively differentiate from the accumulated NCPs (GFP, green) in a spatially and temporally controlled manner (Figs. 2E, F and S3A–C). Immunostaining against RFamide also revealed that at later time points (TP6B), NCPs in the bud differentiate into RFamide-positive neurons (Figs. 2G, H and S3D–F). Based on their emergence in the proximal prospective foot region at later stages of the bud, this is consistent with the appearance of the N1 ganglion cell population in the adult polyp^19^ (Fig. 1E, H).

To follow the formation of neuronal circuits from these emerging neurons, we visualized the development of individual neuronal populations in previously established transgenic animals expressing GFP driven by population-specific promoters (see “Resources table” in “Materials and methods”)^24,25^. The first N6 neurons appear early during bud development (TP2, Fig. 2I), rapidly expand in number in the following 30–40 h to generate, in later bud development stages, a dense interconnected network at the hypostome region (TP6; Fig. 2J–L), highly similar to the adult polyps’ N6 circuit (Fig. 1E, G). Notably, the first sensory neurons within the N6 circuit appear only late, by TP4-6 (Fig. 2F, K, L). In contrast to the N6 population development, which spans the bud’s mid-developmental period, the N1 population begins to form with a substantial delay. The first N1 neurons appear only in the late developmental stages (TP5, Fig. 2M) in the region of a bud most proximal to the parental tissue and always on the aboral side oriented on the parent’s body axis. In the following 30 h, similar to N6 neurons, N1 neurons expand in number and form a dense and interconnected network in the foot region of the bud (Fig. 2N–P), reminiscent of the N1 circuit architecture in adult polyps (Fig. 1E, H).

While the N6 and N1 populations are located in the ectoderm and restricted to the extremities of a polyp (Fig. 1E), the neuronal population N4 forms a nerve net in the endoderm that extends throughout the entire polyp, including the gastric region and the budding zone^24^ (Fig. 1E, F). Does the development of the N4 circuit in the bud follow the same principles as the N6 and N1 populations? Observations on transgenic polyps expressing GFP under the control of an N4-specific promoter (*HVAEP_G008286*)^24,34^ revealed a surprising reorganization of this network during bud outgrowth. First, N4 neurons appear to become less dense at the emerging bud evagination (TP1; Fig. 2Q). Over time, a homogeneous network of N4 cells forms in the endoderm throughout the growing bud (TP2-5; Fig. 2R–T). Intriguingly, this N4 network in the bud appears to be interconnected with the parental N4 network as long as the bud is not separated from the parental animal. In conclusion, nervous system development in the emerging bud is initiated by NCP immigration and follows a specific temporal and spatial pattern.

### The neuronal control of mouth opening capacity develops stepwise and involves the N4 and N6 populations

**Fig. 3:**
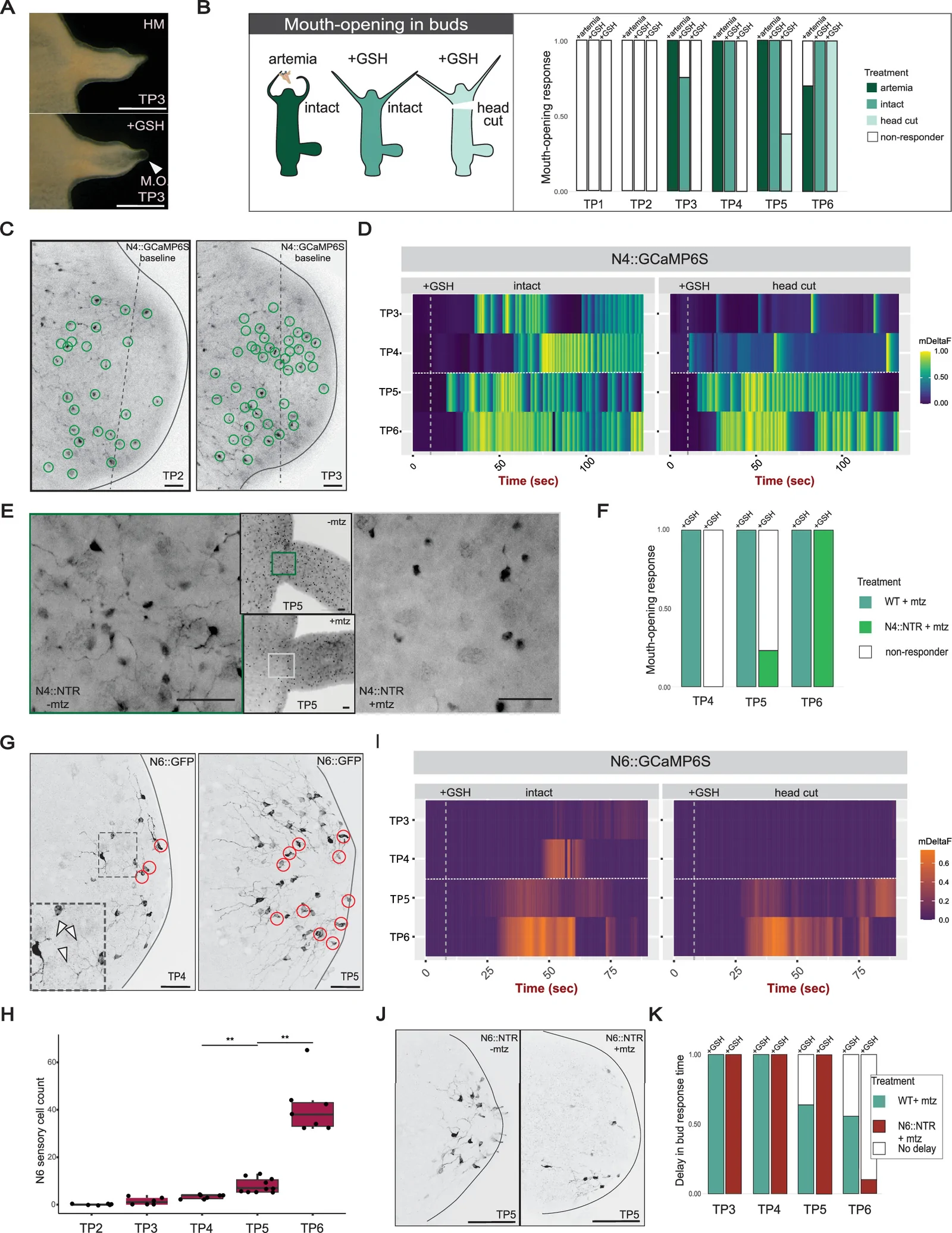
Emergence of the multi-modal control of mouth opening behavior in bud development through sequential formation of N4 and N6 networks. **A** In bud development, the first mouth opening (M.O., *arrowhead*) in response to GSH occurs at TP3. **B** M.O. reflex in the bud is partially dependent on the parental polyp. *Left panel*: experimental set-up scheme. M.O. in a bud can be elicited by GSH administration or presentation of a single prey item (*Artemia*) to the parental polyp. Decapitation of the parental polyp modulates the bud’s response to GSH. *Right panel*: While the first M.O. in buds in response to GSH administration occurs at TP3, feeding a single *Artemia* to the parent directly can also cause a M.O. response in a bud from TP3. In contrast, removing the parent head shifts this initial M.O. to a later stage, TP5 (*n* = 10–16 for each treatment at each time point of bud). **C** Active neurons of the N4 network during a baseline event (without any stimulation) in the same developing bud at TP2 and TP3 show an increased density of active neurons at the distal end of the bud**. D** Spike-train analysis comparing the influence of GSH on the N4 population activity reveals distinct responses based on the presence or absence of the parent head across bud developmental stages. The response of N4 at TP3 and TP4 seems to be dependent on the presence of the parent head. Each row represents the cumulative activity of several neurons in the bud region of one animal with a distinct bud stage (*n* = 3–10 buds for each time point). **E**, **F** Inducible ablation of the N4 population in a budding polyp prevents the bud’s M.O. response to GSH in the early stages of development. **E** The parent-bud junction with a representative TP5 bud. Neurite connections are lost within the N4 network, resulting in a disconnection between the parent and the bud. **F** The fraction of buds responding to GSH. Note that in N4-ablated polyps, buds at TP4 and TP5 do not respond to GSH. Only at TP6, the M.O. response of a bud becomes fully autonomous (*n* = 6–13 for each time point and treatment). **G**, **H** Sensory neurons from the N6 population first appear at TP3 and significantly expand at TP5 compared to TP4 at the tip of the growing bud hypostome; Kruskal–Wallis, *p* = 0.000001652; pairwise two-sided Wilcoxon rank-sum with Holm correction (* *p* < 0.05; ** *p* < 0.01). *n* = 6 (TP2), 6 (TP3), 7 (TP4), 10 (TP5), and, 7 (TP6). Close-up highlight in (**G**) points out gaps between neurites of adjacent cells in the TP4 bud; *arrows* point to neurite endings. **I** Spike-train analysis of N6 population in the developing bud at different time points in response to GSH. The comparison between buds of intact polyps (*left panel*) and decapitated polyps (*right panel*) shows TP5 to be the first stage where N6 cells can respond to the stimulus. Spiking activity of N6 neurons in the buds at TP3 and TP4 in intact animals occurs only after a mouth-opening event and is therefore not involved in the bud response. Each row represents a single animal with a distinct time point. *n* = 4–5 for each time point. **J**, **K** Induced ablation of N6 population in budding polyps results in buds with a significantly delayed response time compared with that of their parent upon GSH addition. **J** Representative TP5 buds before and after ablation. In the buds of ablated polyps, the N6 population is dramatically reduced in size and connectivity and contains no sensory neurons. **K** N6-ablated buds in TP3, TP4, and TP5 all show significant delay in the response time relative to that of the parent, while TP6 shows a much faster response to GSH. *n* = 5–10 for each time point and treatment. Images from representative polyps, scale bar 100 µm (**A**) or 50 µm (**C**, **E**, **G**, **J**).

To examine when the emerging nerve net becomes active and functional during bud development, we investigated neural activity in distinct populations using calcium imaging, cell ablation, and behavioral assays. As part of their complex feeding behavior^35^, adult polyps demonstrate a stereotyped mouth opening response when presented with an *Artemia* prey or exposed to reduced glutathione (GSH). Surprisingly, long before detaching from the parent, a bud is already able to open its mouth (Fig. 3A, B; Supplementary Videos 1 and 2). The mouth opening behavior can first be detected in a bud at TP3 and is then consistently observed during the further stages of development (Fig. 3B). Intriguingly, this response can be triggered not only by exposing budding polyps to GSH, but also by presenting prey only to the parental polyp (Fig. 3B), suggesting a role of the parental neuronal network in eliciting the response in the bud. To test this parental influence, we removed the parental polyp’s head, thereby removing the neuronal cells that initiate the feeding behavior^24^. To our surprise, the removal of the parental head completely abolished the bud’s response to GSH at earlier developmental stages (TP3-4; Fig. 3B), indicating that the bud is not capable of detecting these food-related stimuli in early development. In decapitated polyps, the first response of a bud to GSH stimulation is observed in a fraction of polyps only at TP5, and only by TP6 did each tested bud consistently respond to GSH in the absence of the parental polyp’s head (Fig. 3B). These observations indicate that the neuronal populations located in the head of a parental polyp control the mouth opening circuits of the bud, especially in the earlier stages of its development.

As shown above, N6 neurons are already present very early in the bud (TP2, Fig. 2I), and are restricted to its hypostome, similar to the N6 population in the parental polyp, with no physical connection between the parental and bud N6 populations. Which neuronal networks are involved in the long-range communication between the circuits in the parental head and the neurons in the bud’s hypostome? If one considers the distribution pattern of the neurons in the adult polyp (Fig. 1E–H), only a few populations appear as suitable candidates to conduct the signal through the parental polyp to the bud’s hypostome.

Our previous study identified that the N4 neuronal network in the endoderm (Fig. 1E, F) plays a critical role in the feeding reflex, acting in coordination with ectodermal N6 neurons to control feeding behavior^24^. The N4 network contributes to the coordination and execution of the feeding reflex, including mouth opening, likely through integration of signals through tissue layers^24,31,36^. We have also shown that this population is present in the bud from the very onset of bud formation (Fig. 2Q–T) and is connected to the parental N4 network, making it a promising candidate for mediating the interaction between the parental head and the bud’s hypostome. Deeper analysis using immunohistochemistry revealed, remarkably, that the density of N4 neurons at the distal end of the bud increases significantly from TP2 to TP3 (Fig. S5B), coinciding with the first observed mouth opening at TP3 (Fig. 3B). To determine if the increased number of neurons was functionally integrated into the N4 network, we utilized a transgenic line expressing GCaMP6s under an N4-specific promoter (*HVAEP_G008286*). We used calcium imaging to detect the first spontaneous, stimulus-independent activity of individual N4 neurons and mapped their spatial distribution at single-cell resolution. Our analysis over a single baseline event revealed that the density of spontaneously active N4 neurons at the distal end of an emerging bud was twice as high at TP3 as at TP2 (Figs. 3C and S5A). Therefore, the accumulation of N4 neurons in the head of the growing bud, supported by both immunostaining and activity mapping at the bud tip, correlates with the initiation of the bud’s mouth-opening capabilities at TP3 (Fig. 3B). Administration of GSH and tracing the spiking frequencies of N4 at the population level revealed an increased spiking response across developmental stages in the buds TP3-6 of intact polyps (Fig. 3D, *left panel*; Fig. S5C, *upper panel*). However, at TP3 and TP4 (Fig. 3D), and to some extent at TP5 (Fig. S5C), the responsiveness of N4 neurons is dependent on the presence of a parental head (Fig. 3D). In decapitated polyps (Fig. 3B), N4 neurons in the bud lack increased activity in response to the GSH stimulus and rather show only isolated spikes (Fig. 3D, *right panel*; Fig. S5C, *lower panel*). The absence of continuous activity before TP5 in decapitated polyps (Fig. 3D) provides a mechanistic explanation for the inability of the bud to open its mouth in cases where the parental head is removed (see Fig. 3B). Therefore, expansion of the N4 population in the bud’s distal region seems to be an integral component for the emergence of the mouth opening behavior of the early buds, though in a parent-dependent manner.

To test whether the N4 network is necessary for the coordination of the mouth opening response between the parental polyp and the bud, we ablated the N4 population in both. Overnight incubation of animals that constitutively express nitroreductase (NTR) under the N4-specific promoter (*HVAEP_G008286*) in 10 mM metronidazole (Mtz) leads to a conspicuous disintegration of the N4 network, as indicated by the disappearance of neurites (Fig. 3E). The effectiveness of this method on the same lines has been demonstrated earlier^24^. Most buds with an ablated N4 population cannot open the mouth until TP5 following GSH administration (Fig. 3F), despite the presence of an intact head of parental polyps (compared to Fig. 3B). This provides evidence for a role of the N4 population in the communication between the parental polyp and the bud at the early bud development stages (TP3-4). Intriguingly, some of the buds at TP5 and all TP6 can respond to GSH by mouth opening, despite the absence of N4 cells (Fig. 3F). This suggests the involvement of another neuronal population in establishing an independent responsiveness to the feeding stimuli in the bud. Previously, we showed that the ectodermal N6 circuit is involved in the feeding behavior associated with sensory detection or stimulus-evoked responses. Sensory neurons of the N6 subpopulation are the first to respond to the GSH stimulus, followed by N6 ganglion neurons, and eventually activate the endodermal N4 neurons^24^. This prompted us to investigate the role of N6 in the emergence of the bud’s autonomous mouth opening behavior. Numerous N6 neurons are already present in the bud’s hypostome by TP4 (Fig. 2E, F, K, L). However, the transition to TP5 is marked by a substantial increase in the number of sensory neurons within this subpopulation (Figs. 3G, H and S6B), which likely enables the bud to directly respond to feeding stimuli. This was supported by our observation of the emerging N6 activity using calcium imaging (Figs. 3I and S6A). A transgenic line expressing GCaMP6s under an N6 promoter (*HVAEP_G017227*) was used for this purpose. In intact polyps, the first GSH-induced activity in N6 neurons is detected at TP3-4 (Fig. 3I, *left panel*; Fig. S6A, *left panel*). Since the number of sensory N6 neurons at these stages is still very low (Fig. 3H) and mostly ganglion neurons constitute the N6 population (Fig. S6B), its activity is likely not driven by direct GSH detection and is rather an aftermath of the parent-induced N4-mediated mouth opening of the bud. In fact, in these cases, the mouth opening initiates even before the onset of the neuronal activity (Fig. S6A). Removal of the parental head suppresses this early activity of N6 neurons in TP3–TP4 buds, with direct GSH-induced activity emerging only in TP5–TP6 buds (Fig. 3I, *right panel*; Fig. S6A, *right panel*). Therefore, while the early buds rely on the N4 network for a parent-dependent mouth opening, the later stages seem to rely on the development and functionality of the N6 network. To prove the role of N6 neurons in the emergence of mouth opening control in a bud, we performed an ablation of the N6 circuit using a transgenic line that constitutively expresses nitroreductase (NTR) under the N6-specific promoter (*HVAEP_G017227*). Similar to N4, elimination of N6 neurons caused a rapid disintegration of the emerging N6 network (Fig. 3J) and consequently a temporal delay in the onset of mouth opening, even in the later stages, such as TP5, where the first independent response is expected, indicating an extended reliance of the bud’s mouth opening behavior on the parent (Fig. 3K). Taken together, these findings indicate that the formation of functional N4 and N6 networks occurs in a temporally controlled manner, where N4 is fully assembled at TP3 while still being dependent on parental input and N6 around TP5. By TP6, both networks are fully assembled and independent, enabling the multi-modal control of mouth opening, allowing the bud to perform its feeding response independently from the parent.

### The rhythmic contractile behavior of the bud depends on the N1 population

**Fig. 4:**
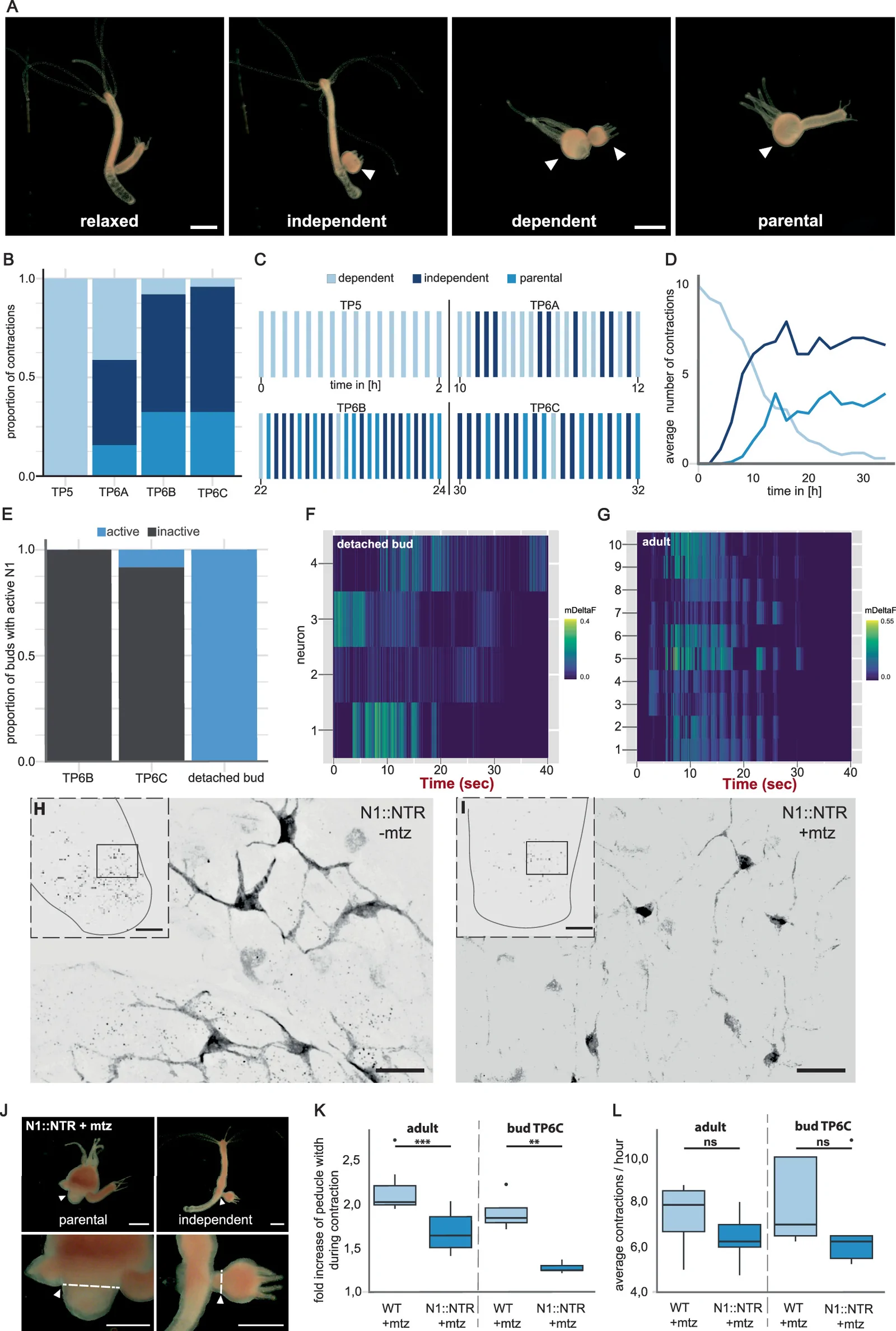
Rhythmic contractile behavior in a developing bud is modulated by the N1 population. **A** Three different contractile behaviors of parent and bud can be observed in budding polyps that carry well-developed buds (TP5-6). Note that a full contraction results in a dramatic shortening of the entire body column of a polyp or bud, and in the thickening and shortening of tentacles. Scale bar: 500 µm. **B** Proportion of bud’s independent contractions and parental contractions increases while dependent contractions decrease throughout bud development, starting from TP5 (*n* = 5). **C** Actogram of one budding polyp monitored over 32 h of late development and progression from TP5 to TP6C, displaying the different contraction behaviors in 2-h intervals across late developmental stages. **D** Average hourly number of dependent contractions decreases, independent and parental contractions increase over time, starting observation from TP5 (*n* = 5). **E** Ca^2+^ activity in N1 neurons was first observed as sporadic localized activity of a single neuron in TP6C and became evident in all buds examined as soon as they detached from the parent (*n* = 5–12). **F** Single-neuron tracking of freshly detached buds from transgenic polyps expressing GCaMP6s under an N1-specific promoter. In an exemplary bud, four N1 neurons exhibit asynchronous activity during contractile behavior. **G** Single-neuron tracking of adult polyps expressing GCaMP6s in the N1 population; N1 neurons in an exemplary adult polyp whose detached bud on (**F**) exhibits synchronous activity during contractile behavior. More replicates are presented in Fig. S7. **H**, **I** Neurite connections of GFP-positive N1 neurons in the foot of an adult *Hydra* are degraded because of Mtz treatment and N1 ablation. Scale bar- overview: 100 µm, close-up: 30 µm. **J** N1-ablated budding polyp with a still attached TP6C bud demonstrates atypical contractile behavior, evident by the inability of the peduncle to contract (compare to **A**). Scale bar: 500 µm. **K** Induced N1 ablation significantly reduces the average fold increase of the peduncle width during one contraction in both adults and TP6C buds; one-sided Mann–Whitney *U* Test, *p* = 0.0009872, 0.007937. **L** Ablating N1 does not significantly reduce the average number of contractions in both adult and TP6C buds; Wilcoxon test. *n* = 8 (adult, WT control), 9 (adult, N1-ablated), 5 (TP6C bud, WT control) and 5 (TP6C bud, N1-ablated) for (**K**, **L**).

In addition to stimulus-evoked behaviors such as the feeding behavior, *Hydra* also exhibits spontaneous behaviors, including recurrent contractile activity^19,30,37^. Initial observations on the emergence of rhythmic contractile behavior in the developing bud revealed three distinct types of contractions that can be consistently observed in budding polyps (Fig. 4A; Supplementary Videos 3 and 4), and have, similar to the mouth opening, both parent-dependent and parent-independent aspects. Dependent contractions (DC) are characterized by the simultaneous contraction of both the parent and the bud and are the only contractions observed during early bud development (till TP5, Fig. 4B). From TP6A onward, we consistently observed independent contractions (IC, Fig. 4A), where the bud contracted without any visible involvement from the parent. Parental contractions (PC) without participation from the bud also became evident during TP6A (Fig. 4A, B). Monitoring the contractile behavior of several individual budding polyps throughout the final four developmental stages of their buds (*n* = 3, TP5-TP6C in total spanning about 30 h, Figs. 4C and S7A) revealed an inverted relationship between the DC and IC frequencies. With progression towards the TP6C stage, the dependent contractions virtually disappeared, while the majority of the contractions displayed by a bud were independent. Taken together, these results suggest an increase in functional independence and asynchrony between the bud and parent.

Earlier studies have indicated that the neuronal population N1, composed of Hym176/KVamide- and RFamide-positive ganglion neurons located in the ectoderm of the polyp’s peduncle, is essential for the polyp’s regular contraction pattern^20,32,38,39^. Staining against RFamide revealed the first isolated N1 cells at the bud’s base in TP5 (Fig. 2M), which increased in number and size by TP6C (Figs. 2G, H, N–P and S7A–C). After bud detachment, RFamide-positive N1 neurons were comparably large and formed a dense network similar to the N1 circuit in an adult polyp (Figs. 1H, 2P, and S7B). To analyze the activity of the emerging N1 network, we used Ca-imaging utilizing a transgenic line that expresses GCaMP6s under an N1-specific promoter (*HVAEP_G016171*; see “Transgenesis and constructs” in “Materials and methods”; Figs. 4E–G and S7E). Surprisingly, we could not detect calcium signals in N1 neurons in buds until very late developmental points. In fact, no spiking neurons were detected even in the TP6B bud (Fig. 4E), and individual active neurons were visible only in some TP6C buds (Fig. 4E). Later, after the detachment of the buds, the activity of N1 neurons was consistently detected in their peduncles (Figs. 4F, G and S7E, *upper panel*). Although these N1 signals in the bud were clearly less synchronous than in the adult polyps (Figs. 4G and S7E, *lower panel*), they were associated with the bud contraction, as in the adult individuals, implying that N1 neurons in the late buds already contribute to the contractile activity. We noted, however, that numerous buds capable of contraction became detached upon the preparation of the TP6C samples for imaging or right at the beginning of the calcium recording, possibly due to external stress. These observations suggest that the fraction of TP6C buds that contain a mature, active N1 circuit and are able to contract is in fact higher than measured (Fig. 4E, *middle bar*), and that N1 neurons are highly active by TP6C. Taken together, our findings indicate that the N1 circuit is fully assembled (Fig. 2P) and active (Figs. 4F and S7E) only at the very late bud developmental stage, TP6C, and its activity pattern remains poorly synchronized even upon bud detachment. Notably, the first IC already emerge in the TP6A buds (Figs. 4C and S7A), i.e., about 20 h earlier. Hence, although our results point to the contribution of N1 to the emerging contractile behavior of a bud, N1 alsone is not fully responsible for the independent buds’ contractions, which might be dependent on another neuronal circuit.

To further investigate the role of the N1 population in contractile behavior, we ablated N1 neurons in transgenic *Hydra* polyps specifically expressing the NTR-GFP cassette under the Hym-176D promoter (*HVAEP_G016171*; see “Transgenesis and constructs” in “Materials and methods”) in N1 neurons (Figs. 4H, I and S7). Similar to N4 and N6, treatment with Mtz resulted in conspicuous disintegration of the N1 network, particularly due to fragmentation and retraction of the N1 neurites (Fig. 4H, I). Although the contraction frequency in late TP6C buds and adult animals was not affected by N1 ablation, the contractile behavior was altered, as the peduncle no longer fully contracted (Fig. 4J–L). At the same time, the upper half of the body displayed typical contraction behavior (evident in body column shortening, lateral expansion, and tentacle retraction, Fig. 4J). Taken together, our observations provide evidence that the maturation of the N1 circuit—increase in neuronal density and neurite complexity (Figs. 2M–P and S7A–C), and emergence and gradual synchronization of its activity (Figs. 4F and S7E)—are essential for the emerging independent contractile behavior of the bud. Since this circuit appears not to be fully mature by the time of the first IC’s appearance (TP6A), the independent contractions might not be solely driven by N1 activity. This aligns with earlier reports showing that the contractile pattern of a polyp is established through interactions between several neuronal populations^20,28,34^. On the one hand, other Hym-176-positive neurons in the bud’s peduncle, which were not genetically labeled in our study, might interact with the Hym-176D-positive population. Additionally, based on our previous study^19^ and earlier reports, we hypothesize that the pacemaker neuron population N2 located in the head of the polyp (Fig. 1E) might also gradually appear in the bud’s distal tip, mature, and trigger the contractile pattern, while N1 in the peduncle ensures the contraction uniformity.

## Discussion

We previously demonstrated that the nervous system of *Hydra* is composed of distinct neuronal populations that have different functions in sensory input and motor output, interact with each other, and generate internal states in a polyp^19,24,31^. During embryonic development, *Hydra* displays a rapid nerve net development, characterized by an activity-dependent assembly, which is sensitive to environmental cues^25^. This process is reminiscent of how functional nerve nets emerge during a short period of embryonic development or organogenesis in other animals^40–42^. However, one of the fascinating aspects of *Hydra* biology is that the polyps can also reproduce asexually through budding^13,43^. Hence, a growing bud in an asexually reproducing polyp represents another remarkable paradigm for the assembly of functional nerve nets. During the budding process, the entire architecture of the parental polyp is likely duplicated in a short time. It endows a freshly detached new polyp with a full repertoire of behaviors. How can a new, fully functional nerve net develop through replication, following the blueprint laid down by the existing parental nervous system?

**Fig. 5:**
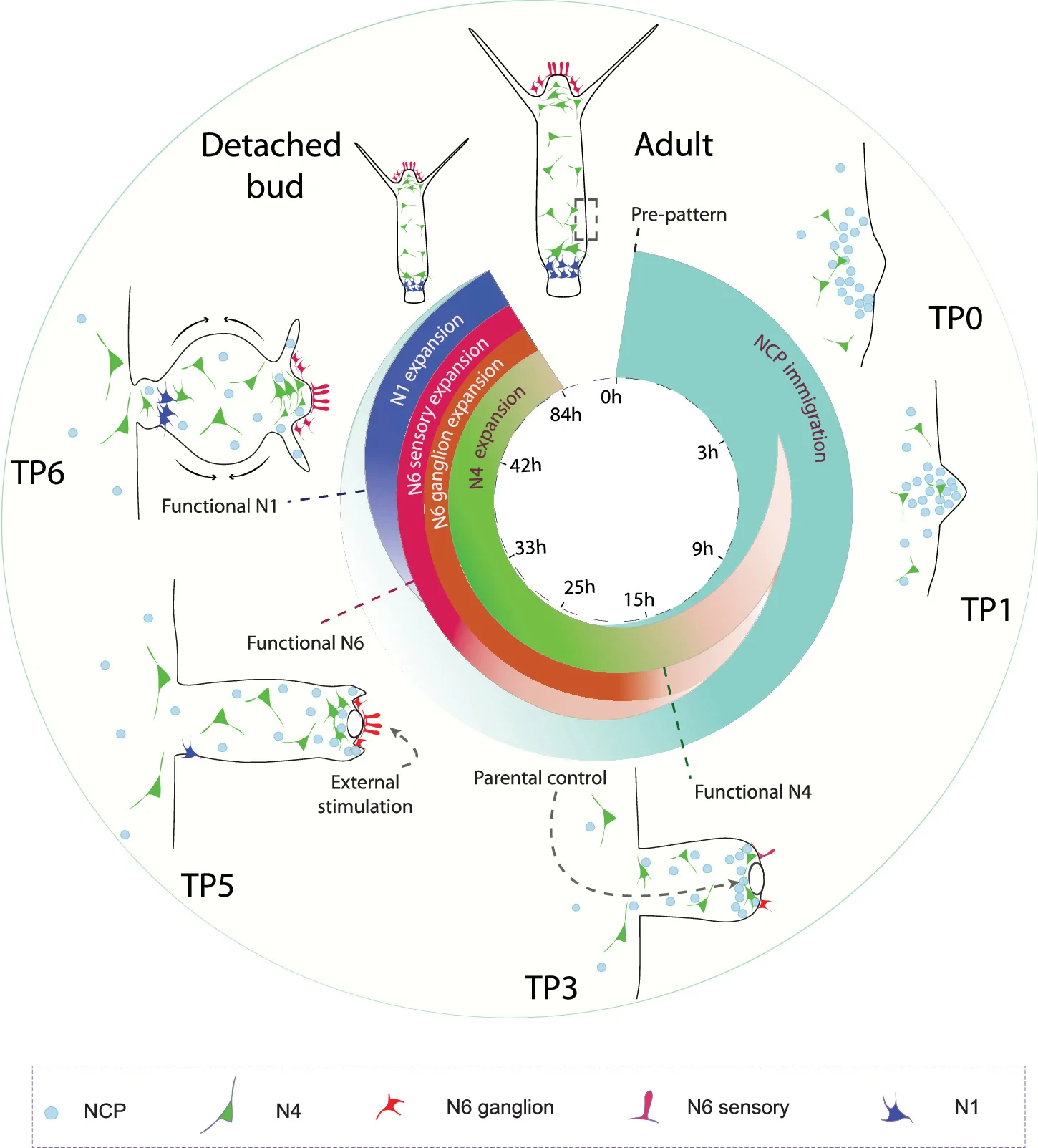
Model - Stepwise replication of a functional neuronal network in the developing *Hydra* bud. Over about 84 h of development, an emerging bud progresses through several critical phases of nerve cell population emergence, activity onset, and behavioral coordination. The bud’s behaviors, initially strongly dependent on the parental polyp, become fully autonomous by the moment of bud detachment. By the time of detachment, the bud possesses all the complete neuronal circuits and displays a behavioral repertoire characteristic of an adult polyp.

Here, we unveil critical principles essential for the replication of this nervous system. First, we show that committed precursor cells are recruited to the site where the new neural network is formed (Fig.5). From this pool of precursors, step-by-step populations of specialized neurons responsible for specific behaviors such as mouth opening and body contractions develop. Surprisingly, while head formation in regenerating *Hydra* has been reported to show a gradual patterning from a mixed pool of tentacle-specific and head-specific neurons, a developing bud appears to contain pre-patterned clusters of these distinct neuronal types^44^. Our observation that NCP accumulation precedes distinct nerve cell differentiation in a spatially controlled manner is consistent with and may underlay the pre-patterned emergence of neuronal subpopulations during budding. The mechanisms that guide the directed migration of neuronal precursors in *Hydra*, however, remain poorly understood. It is plausible that local production of some morphogens in the emerging bud tip^45^ provides the attractant signal for the NCPs and establishes their specific spatial pattern along the axis in the growing bud. Such a mechanism would ensure that the neuronal architecture in the bud faithfully mirrors the parental template and consequently preserves the behavioral repertoire of the organism. We demonstrate a robust developmental program, in which the complexity of an adult neural network, composed of various neuronal populations involved in diverse behaviors, is reliably reproduced during bud development, following a spatiotemporal order.

We analyzed three neuronal populations (N1, N4, N6) essentially involved in two fundamental behaviors, mouth opening and spontaneous body contractions, both vital for the polyps^20,24,27,46^. Using transgenic activity-reporting and cell-ablation lines for these defined neuronal populations associated with well-characterized behaviors^24,31,32^, we could perform a detailed maturation analysis to identify the key steps involved in duplicating an existing neural network at the functional level. The formation of the nervous system in a developing bud occurs in three distinct phases (Fig.5). First, NCPs undergo a pre-patterning phase. Second, NCPs sequentially differentiate into distinct neuronal subtypes, assembling across expanding morphological domains. Third, populations of newly formed neurons expand and increase in density, enabling their associated functions to emerge. Notably, all populations we have studied here exhibit a density-dependent circuit functionality, where a certain neuronal density must be reached in the emerging bud domain for their respective behavioral outcomes. In the case of the N4 population, we could demonstrate that the mere presence of a few active cells on the early bud head (TP2) was not sufficient for the parent-dependent mouth opening function. Moreover, the GSH-sensing capability to initiate mouth opening independently, associated with N6, was also reliant on a dense network of sensory and ganglion cells (TP5). In essence, a denser network indicates a more mature circuit with very close associations of neurites. Similar neuronal density-dependent emergence and maturation of circuit functionality have been observed in dissociated hippocampal cultures from rats, where sparse versus dense plating resulted in distinct network dynamics, with spiking and synchronous bursts strongly dependent on neuronal density^47,48^. However, our in vivo study in *Hydra* takes such density-associated neuronal network functionality a step further by revealing that behavioral emergence follows a stepwise progression from NCP pre-patterning to the maturation of dense, integrated neural circuits in a proliferating animal.

While these neuronal networks in a bud mature during development, the behaviors appear not in isolation, but in tight coordination with the parental nervous system, and a full behavioral independence of the bud is likely established by the moment of its detachment. However, despite the emergence of independent capabilities, parental influence on the behaviors persists, with an overlap with the newly formed behavioral functions of the bud. A logical extension of our work is to ask if the parental network may shape the maturation of neural circuits in the bud. For instance, do the circuits that connect the bud to the parent, such as N4 or N3, fire synchronously throughout development? Or is the maturation to independent circuits in the bud reliant on activity-guided mechanisms through the parental nerve net? Remarkably, the gradual establishment of synchronicity within the nerve nets of *Hydra* has been described in developing embryos^25^ and regenerating aggregates^49^, indicating a central role of synchronization in shaping the developing *Hydra* nervous system. Conversely, one may further ask how a mature neuronal circuit, emerging in the late phases of the bud, resolves conflicting signals from its parent to generate a coherent behavioral output, whether independent or aligned with the parent. Henceforth, while we have laid out a foundation by identifying the key steps through which an existing neural network can be duplicated at the functional level, extending these insights provides an entry point for probing the broader complexities of neural circuit organization and integration.

The sequential formation of neural circuits seems crucial because it imposes order, reduces complexity, and ensures that each step of wiring occurs in the right place at the right time^50,51^. This timing is essential for adapting neural circuits to meet functional demands. Like in *Hydra*, similar principles of guided NCP migration and sequential formation of specific patterns of neural circuits have been demonstrated as critical for the development of the nervous systems in diverse bilateral models like *Caenorhabditis elegans*, *Drosophila melanogaster*, and vertebrates such as mice^9,50,52,53^. The generation of a specific pattern through precursor migration appears to be a universal principle of neurodevelopment, conserved across animal phyla. Thus, the neuronal development and the stepwise formation of neuronal circuits with activity-driven refinement observed in *Hydra*^25,49^ seem to recapitulate a form of neural organization that arose over 600 million years ago during the Ediacaran-Cambrian transition. By revisiting this ancestral blueprint and studying the minimal neuronal requirements, the integration, signal transduction, and coordinated responses in *Hydra* therefore allow us to decode fundamental principles of nervous system development.

## Materials and methods

Resources table

**Table:** 

Reagent or resource	Source	Identifier
Antibodies		
rabbit anti-RFamide	Biozol	T-4322
rabbit anti-DsRed	Takara	632496
chicken anti-GFP	Aves Labs	GFP-1010
Alexa Fluor™ 488 goat anti-chicken	Invitrogen	A11039
Alexa Fluor™ 546 donkey anti-rabbit	Invitrogen	A10040
Alexa Fluor™ 488 goat anti-rabbit	Invitrogen	A11008
Alexa Fluor™ 546 goat anti-chicken	Invitrogen	A11040
Chemicals		
Urethane	Sigma-Aldrich	U2500
Zamboni fixative	Morphisto	12773
Reduced glutathione (GSH)	Roth	6382.1
R2A broth	Neogen	NCM0188A
R2A agar	Roth	CL01.1
pGem-T Easy Cloning kit	Promega	cA1360
Organisms/Strains		
* Hydra vulgaris* strain *AEP*	Prof. Thomas Bosch lab	
* Hydra vulgaris strain AEP (*promoter N6: *HVAEP_G017227::GCaMP6S)* *F1*	Prof. Thomas Bosch lab	
* Hydra vulgaris* strain *AEP (*promoter N6: *HVAEP_G017227::NTR-GFP) F1*	Prof. Thomas Bosch lab	
* Hydra vulgaris* strain *AEP (*promoter N4: *HVAEP_G008286::GCaMP6S) F1*	Prof. Thomas Bosch lab	
* Hydra vulgaris* strain *AEP (*promoter N4:*HVAEP_G008286::NTR-GFP) F1*	Prof. Thomas Bosch lab	
* Hydra vulgaris* strain *AEP* (promoter α-tubulin: *HVAEP_G019559::GCaMP6s*) (pan-neuronal) F1	Prof. Thomas Bosch lab	
* Hydra vulgaris* strain *AEP* (promoter N1: *HVAEP_G016171::GCaMP6s*) F1	This study	
* Hydra vulgaris* strain *AEP* (promoter N1: *HVAEP_G016171::NTRmut-GFP*) F1	This study	
* Hydra vulgaris* strain *AEP* (promoter myc3: *G003730::GFP//*promoter act:*G010650::DsRED*) F1	This study	
Recombinant DNA		
Plasmid- promoter N1: *HVAEP_G016171::GCaMP6s*	This study	
Plasmid- promoter N1: *HVAEP_G016171::NTRmut-GFP*	This study	
Plasmid- promoter myc: *HVAEP_G003730::GFP//*promoter actin: *HVAEP_G010650::DsRED*	This study	
Software		
ZEN blue (v3.6)	Zeiss	www.zeiss.com
Fiji (ImageJ) (v2.14)	NIH	Fiji.sc
Icy (v2.5.2)	Icy	www.icy.bioimageanalysis.org
Adobe Illustrator 2024	Adobe	www.adobe.com
R studio	R studio	www.rstudio.com
Equipment		
Zeiss LSM900	Zeiss	www.zeiss.com
Axio Vert. A1	Zeiss	www.zeiss.com
Colibri 7	Zeiss	www.zeiss.com
38 HE fluorescence filter	Zeiss	www.zeiss.com
5x Plan Apo objective	Zeiss	www.zeiss.com
10x Plan Apo objective	Zeiss	www.zeiss.com
Axiocam 705 mono camera	Zeiss	www.zeiss.com
Channel slide μ-Luer 0.2 mm	Ibidi	cat# 80166

### *Hydra* maintenance

*Hydra vulgaris* AEP polyps were kept under standard cultivation conditions in *Hydra* medium (i.e., CaCl_2_ 0.042 g/L; MgSO_4_ × 7H_2_O 0.081 g/L; NaHCO_3_ 0.042 g/L; and K_2_CO_3_ 0.011 g/L in dH_2_O), as outlined in established protocols^54^. They were maintained at 18 °C in a 12/12-h light-dark cycle, fed *Artemia* nauplii three times a week, and washed 6–12 h after feeding. Prior to any experiments, the animals were starved for 24–48 h.

### Bud development staging

To systematically describe bud development, we generally adopted the bud stage classification^13^, where the 10 distinct bud development stages were identified based on the morphology of the bud anlage, its emerging hypostome, tentacles, and peduncle. Here, TP0 is equivalent to stage 1, or condensation morphology, from the Otto and Campbell study and characterized by a thickening of the parental polyp’s ectoderm (Fig. 1D). TP1 is equivalent to stage 3 per Otto and Campbell, or peak morphology, marked by the appearance of a conspicuous, sharp tip of the evaginating bud anlage. TP2, equal to stage 4, or shield morphology, is characterized by a more square-shaped bud demonstrating a clear proximodistal outgrowth. TP3 is equivalent to stage 5, or bulb morphology. TP4 is similar to stage 6, or rudiment morphology, where conspicuous tentacle primordia become visible. TP5 is between stage 7, or tentacle morphology, and stage 8, or constriction morphology. TP6 is characterized by further distal growth of the bud, elongation of tentacles, and the appearance of constriction at the basal end of the bud, which becomes the bud’s foot. Since some critical events were detected within the TP6 (Figs. 2N–P, 3B and 4B, C), we decided to recognize three stages within the TP6—TP6A (tentacles capable of capturing prey, slight constriction of the proximal bud end), TP6B (prominent constriction between the bud and parental point), and TP6C (thin tissue connection between bud and parental polyp, formation of the bud’s basal disc).

### Transgenesis and constructs

Four previously reported transgenic *Hydra* lines have been used (see Materials table). Additionally, to generate novel transgenic lines, we identified genes strongly expressed in nerve precursor cells (Myc3, *HVAEP*_*G003730*^55^) or exclusively in the neuronal population N1 (Hym-176D, *HVAEP_G016171*) using publicly available resources^17,18^. The promoter sequences of these genes were identified and cloned into expression vectors (Fig. S4). Resulting transgenic constructs were injected into *H. vulgaris AEP* embryos, and stable transgenic lines were established following the standard procedures^54,56^. These three novel lines (*myc::GFP//act::DsRED, N1::GCaMP6s*, and *N1::GFP-NTR*; see “Resources table”) were used to follow the spatial localization, activity, and function of the NCP cells and the N1 neurons, respectively.

### Immunohistochemistry

Whole-mount immunohistochemistry was performed following standard procedures^57^ with slight modifications. *Hydra* polyps were relaxed in 2% urethane (Sigma-Aldrich, U2500), fixed in Zamboni solution (Morphisto, cat#12773), and permeabilized with 0,5% Triton X-100 in PBS. The samples were incubated with primary antibodies (1:500–1:1000 or 1–2 μg/ml) and secondary antibodies (1:1000), embedded in Moviol, and analyzed using confocal laser-scanning microscopy (Zeiss LSM900). The following primary antibodies were used: rabbit anti-FMRFamide polyclonal antibody (Biozol, T-4322), 2 μg/ml concentration in the working solution, rabbit Living Colors anti-DsRed polyclonal antibody (Takara, 632496), 10 μg/ml concentration in the working solution, chicken polyclonal anti-GFP primary antibody (Aves Labs, GFP-1010), 2 μg/ml or 10 μg/ml concentration in the working solution, and secondary antibodies: goat-anti-chicken IgY antibody conjugated to Alexa Fluor™ 488 (Invitrogen, A11039), 2 μg/ml concentration in the working solution, donkey-anti-rabbit IgG antibody conjugated to Alexa Fluor™ 546 (Invitrogen, A10040), 2 μg/ml concentration in the working solution, Alexa Fluor 488 goat anti-rabbit (Invitrogen, A11008), 2 μg/ml concentration in the working solution, and Alexa Fluor 546 goat anti-chicken (Invitrogen, A-11040), 2 μg/ml concentration in the working solution.

### Fluorescence intensity measurement

To quantify the distribution of NCPs in the growing bud, we measured the integral fluorescence intensity of these cells. Raw images taken with a 20× objective at the CLSM were processed using the ImageJ software. Individual channels were processed with the “remove outliers” function with a diameter of 5 pixels to reduce non-specific background noise. The integrated fluorescence intensity within a defined rectangular region extending along the bud axis was measured for each channel using the “plot profile” function. Using the data from these measurements, graphs were generated displaying the integrated fluorescence intensity along the bud’s axis (Fig. 2A–D, *lower panel*; Fig. S1I–L, *lower panel*). To smooth the resulting graphs, a geometric mean of the following 100 values was calculated for each intensity point.

### Nerve network architecture analysis

To quantitatively describe the architecture of the emerging nerve populations, we measured the cell density, number of sensory and ganglion neurons, and number of neurites per neuron on *Hydra* samples stained, embedded, and imaged as described above (see “Immunohistochemistry”). ZenBlue 3.4 (Zeiss) and Fiji software were used for image processing and measurements. The Cell Counter plugin from Fiji^58^ was utilized to analyze the distribution and densities of neurons. To count comparable areas, the head region visible on the images was selected by drawing lines around the region with the polygon tool from Fiji ImageJ (see, for instance, Fig. S5B). We computed the density of neurons per 1000 square micrometers for the densities by dividing the number of cells counted by the area of the head for the selected region.

For counting N6 cells, sensory and ganglion neurons (Figs. 3H and S6B) were manually identified while counting with the Cell Counter plugin. Sensory neurons are characterized by their spindle-shaped somata, a nucleus located within the ectoderm layer, and the presence of apical cilia. Ganglion neurons lack apical cilia, and their bodies with nuclei are located at the base of ectodermal cells, adjacent to the mesoglea^23^. To analyze the neurite properties of the N1 population (Fig. S7D), all primary neurites of 10 neurons per replicate were counted and averaged.

### Mouth opening behavior with GSH or *Artemia* stimulus

To investigate the mouth opening function of the bud, the GSH stimulation assay^24^ was used with slight modifications. An individual polyp carrying a bud was picked randomly from the culture and placed on a plastic Petri dish under a stereo light microscope. The stage of bud development (Fig. 1D) was identified and recorded. After the polyp carrying the bud had relaxed after 3–4 min, the surrounding *Hydra* medium was gently aspirated until only a residual volume of approximately 50–100 μL remained to keep the animal submerged. Then, approximately 1 ml of 10 µM GSH in *Hydra* medium was carefully added over the animal using a glass pipette. Because the residual volume was negligible compared to the added GSH solution, the final concentration of GSH was effectively 10 µM. The tips of the buds were monitored through the stereo microscope for 5–10 min. The bud would be scored as a responder if the tip of the bud was seen protruding during the GSH administration, leading to a small opening of the mouth (Fig. 3A, Supplementary Videos 1 and 2). To study the influence of the parent in this response, the head of the parent was removed by a single transversal cut with a blade, 5–10 min before testing the bud response in the setup described above. To demonstrate the direct influence of the parent on the bud, a single *Artemia salina* nauplius was placed near the tentacle of the parental polyp, and the bud was immediately observed for mouth opening under the stereo microscope. To ensure that the bud’s response was directly from the parent and not because of any secretions during the feeding of the parent, a separate adult animal was placed at very close proximity to the parent and bud under study. If this control polyp responded, although the prey was not presented to it, the entire replicate would be discarded. For each treatment at each time point of the bud, 10–16 replicates were used (Fig. 3B). All the behavior tests were carried out at 18 °C.

### Contractile behavior

To analyze the contractile behavior of *Hydra* polyps and their attached buds, three animals were placed into one well of a 48-well plate, which ensured that the animals could move freely. The contractile behavior of the bud was also studied with animals at the TP5 stage using the same setup. The well was filled with 1 mL of fresh *Hydra* media. The animals were recorded for 36 h at 18 °C with a frame rate of 0.1 frames per second. Contractile behavior that consisted of body column shortening, lateral expansion, and tentacle retraction was scored manually for both the parental polyp and the attached bud. Using these measurements, the contraction frequency per hour was calculated for 3 replicates of parent-bud pairs.

### N4 ablation and mouth opening

To investigate if N4 was involved in the bud’s early mouth-opening response under the parental influence, animals carrying the GFP-NTR cell suicide cassette under the control of the N4-specific promoter were used (N4::NTR-GFP)^24^. Animals carrying early buds with stages ranging from TP0-TP3 were transferred into a six-well plate and kept in 9 ml of 10 mM metronidazole (Mtz, Sigma 443-48-1) solution in the dark for 16 h and at 18 °C. Such stage selection ensured that we obtained a range of early- and late-stage buds by the time the Mtz treatment was over. In this way, we could get TP4-TP6 buds, which provided us access to both the dependent and independent stages of mouth opening (Fig. 3B). Between 6 and 13 replicates were used for each time point of both Mtz-treated and control animals. After the treatment, the animals were screened with an Axio Vert. A1 (Zeiss) microscope, utilizing the Colibri 7 LED light source and the 38 HE fluorescence filter at 10×. If GFP-positive cells were visible only as small remnants with disrupted neurite connections or completely absent (as in Fig. 3E), the buds were characterized for their developmental stage at that moment and used for behavioral assays as described above. Wild-type controls were treated the same way for comparison. Some animals were also picked for immunohistochemistry to demonstrate ablation and disconnection of the network between the parent-bud junction.

### N6 ablation and mouth opening

To demonstrate that the presence of N6 contributes to the initiation of the independent mouth opening in a developing bud (Fig. 3J, K), we used animals carrying the GFP-NTR ablation cassette under the control of the N6-specific promoter (N6::NTR-GFP)^24^. The selection of animals and their treatment with Mtz was as described above for the N4 ablation experiment. However, the analysis could not simply rely on the presence or absence of a response to GSH since N6 is only restricted to the hypostome of the adult or the emerging bud tip, and the neuronal population connecting the parent and bud, i.e., N4, would still be present, resulting in a parent-dependent response for the bud. We addressed this by quantifying the delay in the mouth opening response of the bud compared to its parent. A dependent response of a bud would be either slower or about the same time as that of the parent, while an independent one would be either faster or occur at the same time as the parent. Therefore, an established protocol^24^ was employed here to record mouth opening in both the parent and the bud during GSH administration in customized chambers with a height of 0.4 mm. The recordings were done using M3C Wild binocular microscopes and Axiocam 208 color (Zeiss), with a frame rate of 0.5 frames per second. The frame at which the first mouth opening occurred for each bud-parent pair was identified from the recordings in a blinded study by assigning a random 6-digit/letter code for each recording (see Supplementary Data 1). The proportion of buds responding after their respective parents, or the delayed response, was recorded as an indicator of parental dependence, in contrast to an independent response. Wild-type animals used as controls were treated the same way for comparison. For each time point and treatment (Mtz vs HM), 5–10 replicates were used. Some animals were also picked for immunohistochemistry to demonstrate ablation, where sensory and ganglion neurons were ablated and neurite connections disrupted.

### N1 ablation and bud contractions

To investigate the role of N1 in modulating contractile behavior, animals carrying the NTR ablation system under the control of an N1-specific promoter (see “Transgenesis and constructs” in “Materials and methods”) were used. Five animals were selected, and the presence of GFP-positive cells was confirmed. After transferring the animals to a six-well plate, they were treated with 10 mM Mtz solution and kept in the dark for 16 h at 18 °C. The next day, the animals were placed in a custom-made chamber with a height of 7 mm and a volume of 1.5–2 mL Mtz, ensuring unrestricted movement of the animals. The contractile behavior of N1-ablated and wild-type control animals was recorded for 4 h. Contractile behavior was scored manually using ImageJ according to previously established criteria (see “Contractile behavior”). Using these measurements, the number of contractions per hour was calculated (Fig. 4A–D). Similar to those mentioned in the contractile behavior analysis, the fold change of the peduncle width during contractions was measured using ImageJ (Fig. 4J–L). The length was defined using a self-defined scale. To confirm the ablation of N1, animals were relaxed with ice-cold urethane 2% for 2 min and fixed with Zamboni immediately after treatment with Mtz. Then, immunohistochemical detection of GFP using specific antibodies (see “Immunohistochemistry”) was performed, and neurite connectivity was investigated microscopically.

### Calcium imaging

In order to investigate the neuronal activity of the newly formed neural circuits in the developing bud, the animals were placed in commercially available channel slides with a height of 0.2 mm and 0.55 mm width or a height of 0.1 mm with a width of 1 mm. After being transferred into the chamber, animals could adapt for 10 min and 2 min to the blue LED excitation light. Recordings of calcium activity were performed with the Axio Vert. A1 (Zeiss) microscope, equipped with a Colibri 7 LED light source, the 38 HE fluorescence filter, 5x and 10x Plan Apo objectives, and an Axiocam 705 mono (Zeiss) camera. Recordings were performed either with an LED intensity of 14–18% and a frame rate of 24.45 frames per second. Then the recordings were processed using the Zeiss Blue 3.4 software and converted to 8-bit using ImageJ. The acquired file was then used for analysis, either with ImageJ for tracking all neurons in a region of interest or using ICY software with the EMC^2^ protocol^59^ for tracking single neurons. Customized R scripts were used to normalize the data before visualization of the single-cell tracking^60^. For each tracked neuron, a fluorescent intensity trace was extracted from the ICY tracking plugin. Prior to normalization, the fluorescence signal was corrected for local background. Then these tracks were normalized to the initial fluorescence intensity (baseline fluorescence) of the cell to account for differences in expression level and illumination within the neurons using the formula delta*F*/*F*_0_, where *F*_0_ is the mean baseline fluorescence of each neuron.

### Tracing active N4 neurons over development

Parental N4 could influence the mouth opening of the bud from TP3, while the activity of N6 appears independent of that of N4 until TP2. To determine and hence compare the functional neurons present during these two stages, we performed calcium imaging over a baseline event of the N4 population, visualizing the young buds. For this, we used animals carrying the GCaMP6s reporter under the control of an N4-specific promoter (N4::GCaMP6s)^24^. TP2 buds were imaged first in vivo and then kept at 18 °C for 4–6 h to allow the bud to develop into a TP3. This was confirmed by examining both time points under a light microscope. The buds were collected and placed in the commercially available channel slides with a height of 0.2 mm and a width of 5 mm.

The imaging was conducted until at least two baseline activities were detected in the 10 min recording at 24.45 fps. Later, the event, where the neurons are most clearly visible throughout the entire bud region, was extracted, with no spikes observed before or after the selected event. The selected event duration ranged between 400 and 3700 frames, or approximately 16–151 s. The chosen frame range was then inverted in ImageJ for better distinction of the neurons from the background. This process was repeated for all the replicates before analysis in the ICY software, where the neurons were tracked using the EMC^2^ algorithm^59^. The tracks generated were checked to verify changes in fluorescence and to ensure that tracks were not fragmented for any neurons. The Track Processor Display Number plugin was used to display the number on each marked neuron. Since the goal of this experiment was to locate the active neurons along the bud column, the tracked image sequence was screenshot to save the marked and numbered neurons. Later in ImageJ, a frame was selected where most neurons were marked to acquire the full potential of functional neurons in the bud stage. The sequence was double-checked with the raw recording to ensure the neurons were true. It was also checked that the neurons detected would produce longer and uninterrupted tracks in the ICY Track Manager view to ensure that the number of neurons in our selected frame for mapping was relatively stable throughout its nearest upstream and downstream frames. These quality checks made the cells marked from the selected frame more reliable. To get an estimate of the number of neurons active in the distal versus the proximal end of the bud, a line was drawn in the middle of this final selected frame. 3 such replicates, where the bud tissue had a clear lateral view during the spontaneous activity for both TP2 and TP3 (Figs. 3C and S5A), were analyzed.

### N4 activity in response to GSH

To measure N4 activity and demonstrate the involvement of this population in the parent-dependent mouth opening response of the buds during GSH administration, we once again used animals carrying the GCaMP6s reporter under the control of the N4-specific promoter^24^. A previously established method was used for this analysis^24^, where animals with selected buds were placed in commercially available channel slides with a height of 0.2 mm and a width of 5 mm, and allowed a 10 min adaptation time in the chamber, followed by a 2 min acclimatization to the LED light. Reduced glutathione solution was gently added thereafter, making sure the bud stayed relatively stable in position.

Buds of decapitated polyps were used to show the absence of N4 activation in early bud stages without parental stimulation, while intact animals were used to show changes in N4 population activity during GSH administration (Figs. 3D and S5C). Between 3 and 10 replicates were used for each time point of both experimental and control animals. The recording was carried out with 14% LED intensity and a 10x objective, keeping the distal end of the bud in focus. Since N4 shows synchronous activity patterns in all neurons, we analyzed the population dynamics rather than choosing single neuronal dynamics. As a result, the mean activity of the entire N4 population present in the bud at that moment was measured in ImageJ^58^.

To ensure robust extraction of neuronal activity, fluorescence image stacks were pre-processed in Fiji^58^ using a custom macro. First, brightness and contrast were normalized across the full stack using an auto-enhancement routine (0.35% saturated pixels), followed by application of a uniform LUT. To standardize intensity scaling across recordings, the maximum gray value was set to 255 and the minimum to 245, thereby normalizing signal dynamics and minimizing variability introduced by background fluctuations. For quantification, a full-frame ROI was defined, and mean gray values were extracted for each frame using the ROI Manager multi-measure function. Resulting values were exported as .csv files and subsequently used for downstream analysis of fluorescence activity in R^61,62^. To account for variation in absolute fluorescence levels across recordings, each trace was normalized using a robust baseline (*F*_0_). Normalized activity was then expressed as a fractional change relative to this baseline using delta*F*/*F*_0_. All extracted activity traces (processed data) were cross-checked against raw image data to confirm that signal dynamics were preserved.

### N6 activity in response to GSH

The N6 subpopulation in *Hydra* is restricted to the hypostome region on the ectodermal layer and has two cell morphologies: sensory and ganglion. It functions as the initiator of the chemosensory signal from GSH, with sensory neurons activating first, followed by ganglion neurons to propagate the signal^24^. To measure the activity of these emerging neurons in developing buds upon GSH administration, we used animals carrying the GCaMP6s reporter under the control of an N6 promoter^24^.

Since the first responders were among the TP3s, we studied the neuronal activities of at least 3 replicates between TP3 and TP6 for both the head cut and intact animals (Figs. 3I and S6A). Selected buds were placed in channel slides with a height of 0.2 mm and a width of 5 mm and given a 10 min adaptation time in the chamber, followed by 2–4 min of acclimatization to the laser, which was at 14% intensity. When animals appeared relaxed, with the focus of the 10× objective on the bud tip, 10 µM GSH solution was gently added from one end of the slide and recorded for at least 4 min at a frame rate of 25 fps. The recordings were converted to 8 bits and standardized by extracting 200 frames before adding GSH and 2200 frames after, totaling 2400 frames for each replicate. 2200 of the tracked frames were plotted, as shown in Figs. 3I and S6A.

The neuronal activity in this population is not synchronized like that in N4, as the sensory neurons respond before the ganglion neurons. Therefore, we used a single-cell analysis with the ICY software for analyzing N6 activity. Our goal was to investigate the presence or absence of activity due to the direct influence of the parent. Hence, we selected only one sensory neuron for any given time point of the bud to track. Additionally, at earlier time points such as TP3 or TP4, there was as few as one possible sensory neuron showing activity, which further justified tracking a single sensory neuron across all stages. The neuron was analyzed using the EMC^2^ protocol in ICY^59^, and fragmented tracks were connected. The data underwent quality control by comparing it with the raw data.

### N1 activity during contractile behavior

To investigate the calcium activity of N1 neurons during contractile behavior, animals carrying the GCaMP6s reporter under the control of an N1-specific promoter were used (see “Transgenesis and constructs” in “Materials and methods”). Animals with a TP5-6C-attached bud were transferred into commercially available channel slides with a height of 0.2 mm and a width of 5 mm. Detached buds, however, were too small; hence, they were placed in channel slides that were 0.1 mm high and 1 mm wide to improve focus and resolution. Analysis of the recordings was performed within a period of two well-visible contraction events. 1000 frames from videos containing these events were extracted and further analyzed using the EMC^2^ protocol^59^ in ICY. Utilizing the Track Manager, fragmented tracks were connected, and false or non-specific tracks were fixed. Single neuron activity was further analyzed using customized R scripts (Figs. 4F, G and S7E).

### Statistics and reproducibility

All statistical analysis and plot generation were performed with R^62^ and RStudio^61^. Prior to any statistical tests, normal distribution, using the Shapiro-Wilk test, and equal variances, using Levene’s test, were checked. To estimate linear relationships between different contraction types during development of the bud, a general linear model was calculated. For pairwise comparisons of more than three groups in non-normally distributed data, a Kruskal–Wallis test, followed by a Dunn test with Bonferroni corrections, was performed. If the data were normally distributed and possessed unequal variances, a Welch’s ANOVA followed by a Games-Howell test was conducted. But for normally distributed data with unequal variances, where the cell number data had a lot of zeros in the early stages (N6 sensory neuron counts), a Kruskal–Wallis test followed by a Wilcoxon rank-sum test with Holm correction was used. The same was also used in case of a violation of both normality and equal variance, as in the case of N6 ganglion neuron counts. For comparisons of two independent, non-normally distributed groups, Mann–Whitney *U* tests were utilized. Statistical significance was marked as ns *p* > 0.05; *p < 0.05; **p < 0.01; ***p < 0.001. The number of replicates and the specific statistical test used were documented in the figure legend and again in the methods section. Raw data used for the figures have been provided in Supplementary Data 1.

### Reporting summary

Further information on research design is available in the Nature Portfolio Reporting Summary linked to this article.

## Supplementary information

Transparent Peer Review file

Supplementary Information

Supplementary Data 1

Supplementary Video 1

Supplementary Video 2

Supplementary Video 3

Supplementary Video 4

Reporting Summary

## Data Availability

All numeric data that support the findings of this study, presented in existing plots in the figures, are provided in Supplementary Data 1. All raw imaging associated with these numeric data and data visualizations is available from the corresponding authors upon request. Further information and requests for resources and reagents should be directed to the lead contact, T.C.G.B. (tbosch@zoologie.uni-kiel.de).

## Code availability

This paper does not report any original code. All codes used for analyzing calcium imaging data have been adopted from previously published work (https://github.com/ChristophGW/Cell-Reports_Satiety-Hydra)^24,31^. Adopted codes in this study are also available from the lead contact upon request (tbosch@zoologie.uni-kiel.de).
