# Supplementary material for: In vivo replication of a fully functional nerve net in a clonally reproducing animal
Source: Commun Biol. 2026 Oct 22;9:1389. doi: 10.1038/s42003-026-11184-x (PMC13690700; doi:10.1038/s42003-026-11184-x)
Supplement: Supplementary file 1 [file 42003_2026_11184_MOESM1_ESM.pdf]

nature portfolio

Peer Review File

In vivo replication of a fully functional nerve net in a clonally reproducing animal

Corresponding Author: Professor Thomas Bosch

This file contains all reviewer reports in order by version, followed by all author rebuttals in order by version.

Version 0:

Reviewer comments:

Reviewer #1

(Remarks to the Author)
This paper documents and experimentally analysis the emergence of the different neuronal circuits in the budding Hydra polyp. The paper is a masterpiece in regard to designing the experiments, visualizing the data, explaining the results. It offeres a wealth of new insights into circuit assembly.
My comments for textual improvements:

1.Line 105-115: The use of specific markers for neuron subpopulations should be explained in more detail. From what I understand, Rfamide is expressed in N1, N6, N7. By using this marker how can the authors determine which of these subpopulations they are looking at?

2.line 122: it would help the reader if the authors explain (introduction) the composition of a circuit, like N6, of different neuron types. From l.122 I assume that N6 is composed of at least two types, sensory neurons and ganglion cells (?). Is that true for all circuits? Is it easy to distinguish the two types (morphology?). What are the implications: even though they belong to the same circuit, are these cell types molecularly distinct?

3.line 138-150: the morphogenesis of ectoderm vs endoderm and the mouth in the bud should be explained. At the stages shown in Fig.2, does the endoderm forms a solid mass of cells in the interior of the bud? And the NPCs forming N4 vs N1/N6 form part of the endoderm vs ectoderm? Is it correct that in the parental polyp, the NPCs that will contribute to all of the NPCs entering the bud are considered ectodermal? If so, does that mean that during the early budding process they lose their ectodermal affiliation (because they give rise to ectodermal and endodermal neuron circuits)? How does the enderm become an epithelium, and how does the mouth opening form in the budding polyp?

4.line 171ff: Here some more context of how separate/interconnected the different circuits are would help. My (simplifying) instinct wants to make me believe that the circuits are separate, and responsible for different behaviors. This is obviously not so, but the basics of how N4 is integrated with N6/7 in feeding behavior should be briefly summarized. From what is described here, I assume that N4 is also responsible for mout opening in mature polyps? Is that a bit counterintuitive, given that N6/7 are located right at the mouth?

5.line 198: is it known why MTZ kills only N4 neurons?

6.line 218ff: I come back to the different roles of N4 vs N6 in feeding behavior/mouth opening. My confusion: I assume that “mouth opening” is caused by a specific set of muscles (a ring/sphincter-like array surrounding the mouth?). Do the reported findings mean that N4 and N6 both overlap in this muscle subpopulation? Or do N4 and N6 innervate different muscles, but contraction of either of them cause the same movement (i.e., mouth opening)? This is somehow not plausible.

7.line 274 (related to #5): I see now that MTZ is only part of a more involved method to ablate specific cell populations. It would help to very briefly point out the mechanism.

Reviewer #2

(Remarks to the Author)
Asexual budding or colony building is a central feature of many Cnidarian clades. In such a fashion a fully build animal has the capacity to develop its clone. The Hydrozoan Hydra vulgaris has been widely used for regeneration studies, but also for its capacity to propagate clonally.
In view of tissue generation during clonal reproduction a key question is how specific cell types emerge and how they are integrated into functioning tissues. This question is is particularity intriguing in the case of the nervous system.
The current study follows the emergence of several previously identified neuron types, which are critical for feeding.
They authors show that neural progenitors precede the emergence of neurons; furthermore they observe how the different types emerge and record their activity during the process.

This is overall an timely manuscript, all experiments of high quality. The study focusses on a thorough analysis mostly on cell types required in feeding.
I do not have any major concerns and from what I can see the conclusions draw are in line of the evidence presented.

One point that might be worth considering is that while the early diversification covers on neural progenitors, most/all of the subsequent analysis of neural maturation is focussed on a couple subtypes. This in turn does not include all neurons and generalisations form the observation should be made carefully. I why the authors did not look into the pan-neuronal line, which for hydra exits?

Another minor point might be that Calcium imaging is only the last step in the process of wiring up of circuits ensembles, thus the developmental processes how this occurs and if this process is similar to what occurs during development can not be drawn.

Reviewer #3

(Remarks to the Author)
The study by Sakib et al is very exciting, highly novel and thoroughly performed. It will definitely be interesting to the evolutionary neurobiology, evo-devo, and zoology fields. This manuscripts dissects mechanisms of formation of neural circuits and behaviours under their control in an animal with a very simple nervous system, freshwater polyp Hydra, during its reproduction through asexual budding. Surprisingly, the main principles turned out to be the same as in animals with much more complex nervous systems (e.g. vertebrates) suggesting that these mechanisms are very ancient and highly conserved across the animal evolution.
The experimental procedures are described in detail.

I only have a couple of minor comments:
1. Line 89: When first introducing the new myc3::GFP//act::RFP line, it would be nice to mention what the purpose of act::RFP transgene is, not only myc3::GFP.
2. Line 196: It would be helpful to mention the genotype of the N4 ablation line, otherwise it is unclear why only N4 population disintegrates by the MTZ treatment.
3. Line 689: It would be good to connect the genotypes to the lines described in the text (promoter t12588 is not mentioned in the text)
4. Line 944: “corresponding GFP-positive” – should be “corresponding to GFP-positive”
5. Line 945: red arrowhead seems to be missing at the figure.

I think this manuscript is suitable for publication.

Version 1:

Reviewer comments:

Reviewer #1

(Remarks to the Author)
The authors have revised their manuscript and addressed my questions and comments in great detail. In my opinion, with these added information, the manuscript is ready for publication.

Reviewer #3

(Remarks to the Author)
All my comments have been addressed, thanks!
Just a typo that needs to be fixed: "transgeneis" instead of "transgenesis" on line 289. Also, it will be clearer to say "see Transgenesis and Constructs section in Methods" rather than just "see transgenesis".

Open Access This Peer Review File is licensed under a Creative Commons Attribution 4.0 International License, which permits use, sharing, adaptation, distribution and reproduction in any medium or format, as long as you give appropriate credit to the original author(s) and the source, provide a link to the Creative Commons license, and indicate if changes were made.
In cases where reviewers are anonymous, credit should be given to 'Anonymous Referee' and the source.
The images or other third party material in this Peer Review File are included in the article's Creative Commons license, unless indicated otherwise in a credit line to the material. If material is not included in the article's Creative Commons license and your intended use is not permitted by statutory regulation or exceeds the permitted use, you will need to obtain permission directly from the copyright holder.
To view a copy of this license, visit https://creativecommons.org/licenses/by/4.0/

Manuscript # COMMSBIO-26-2162

Specific changes in the text and figures and comments to the Referees:

Reviewer #1 (Remarks to the Author)

This paper documents and experimentally analysis the emergence of the different neuronal circuits in the budding Hydra polyp. The paper is a masterpiece in regard to designing the experiments, visualizing the data, explaining the results. It offeres a wealth of new insights into circuit assembly.

We thank the referee for the positive and encouraging comments.

My comments for textual improvements:

1.Line 105-115: The use of specific markers for neuron subpopulations should be explained in more detail. From what I understand, Rfamide is expressed in N1, N6, N7. By using this marker how can the authors determine which of these subpopulations they are looking at?

Thank you for raising this important point, which we obviously have not addressed very well in this section. We confirm that RFamide is expressed in multiple neuronal populations (including N1, N6, and N7) and is therefore not population-specific on its own. In our study, the identity of specific neuronal populations was inferred by integrating RFamide immunostaining with spatial and temporal context during budding, as described for *Hydra* neuronal populations. The terminology and spatial location of each population of neurons have already been established (Fig 1D + Supplementary – Klimovich *et al.* PNAS 2020; Siebert *et al.*, Science, 2019), while the populations mapped on the *Hydra* polyp can be found in Fig 1D in Giez *et al.* CurrBiol, 2023, which we have also adopted in this paper in Fig 1E. In this study, the early appearance of RFamide-positive neurons at the hypostome tip is consistent with the localization of the N6 population. The tentacle-specific N7 may only appear by TP5 when tentacle buds appear on the bud's head and is identified in their localization on the young tentacles. The RFamide-positive neurons emerging later in the proximal bud region correspond to the expected position of N1 neurons. We note that we do not observe patterns consistent with N7 localization in these contexts. We have revised the manuscript (see lines:137-139 in the revised manuscript) to more clearly explain that our neuronal population assignments are based on this combined framework rather than RFamide expression alone.

2. line 122: it would help the reader if the authors explain (introduction) the composition of a circuit, like N6, of different neuron types. From l.122 I assume that N6 is composed of at least two types, sensory neurons and ganglion cells (?). Is that true for all circuits? Is it easy to distinguish the two types (morphology?). What are the implications: even though they belong to the same circuit, are these cell types molecularly distinct?

Thanks for the comment. Yes, you are absolutely right. N6 does in fact consist of two types of nerve cells based on morphological differences – sensory and ganglion. However, this is not true for all circuits; aside from N6, only N3 and N4 are known to consist of both sensory and ganglion morphologies. The sensory neurons are mainly distinguished from the ganglion cells based on the projection of their sensory cilia on the epithelium surface.

However, no molecular distinction has yet been reported between the two cell morphologies in cells of any particular neuronal population in *Hydra*. We have added a more detailed background about these cell types in the revised introduction section now (see lines 74-90).

3.line 138-150: the morphogenesis of ectoderm vs endoderm and the mouth in the bud should be explained. At the stages shown in Fig.2, does the endoderm forms a solid mass of cells in the interior of the bud? And the NPCs forming N4 vs N1/N6 form part of the endoderm vs ectoderm? Is it correct that in the parental polyp, the NPCs that will contribute to all of the NPCs entering the bud are considered ectodermal? If so, does that mean that during the early budding process they lose their ectodermal affiliation (because they give rise to ectodermal and endodermal neuron circuits)? How does the enderm become an epithelium, and how does the mouth opening form in the budding polyp?

We thank the reviewer for raising this important point regarding bud morphogenesis and the relationship between ectodermal and endodermal tissues, as well as the origin and fate of neuronal progenitor cells (NCPs).

Bud morphogenesis in *Hydra* has been described in detail in previous studies (Holstein *et al.*, 1991; Otto and Campbell, 1977). During this process, although a pronounced thickening of the endoderm is consistently observed beneath the evaginating ectodermal bud primordium (e.g., Webster and Hamilton, 1972), the endoderm does not form a solid mass of cells at any stage. Instead, both ectoderm and endoderm remain as continuous epithelial layers throughout budding. Importantly, the endoderm of the developing bud is directly continuous with that of the parental polyp, and both share a common gastric cavity (see lines 59–61 in revised manuscript). We agree that this continuity is functionally significant. It provides a structural basis for the extension of the N4 neuronal network from the parental polyp into the growing bud (Fig. 2Q–T), thereby enabling coordination between the hypostomes of the parent and the bud (Fig. 3A–D).

Regarding the origin and fate of NCPs, these cells arise in the ectoderm of the parental polyp from interstitial stem cells (Holstein and David, 1986). They give rise to all ectodermal neuronal subtypes (N1, N2, N3, N6, and N7). In addition, a subset of transient progenitors migrates through the mesoglea into the endoderm, where they differentiate into endodermal neuronal populations (N4 and N5), as well as gland cells (see lines:71-73 in the revised manuscript). The lineage relationships and differentiation trajectories of these progenitors have been described in detail by Siebert *et al.* (2019).

Thus, while all NCPs originate from the ectodermal lineage, their ability to migrate and differentiate into endodermal derivatives reflects lineage plasticity rather than a loss of origin. Both ectodermal NCPs and migrating progenitors share common molecular signatures, including expression of myc3 and myb. We leveraged this feature to generate our transgenic reporter line (Fig. S4), in which GFP expression is driven by the myc3 promoter. Consistent with the endogenous expression pattern of myc3 (Siebert et al., 2019; Lechable et al., 2023), this reporter labels the full population of immature progenitors irrespective of their final localization.

We assume that the question of the Reviewer arises mainly due to the fact that most of the images we present are Maximum Intensity Projections from confocal Z-stacks, which do not allow for perceiving the depth of cells' localization. To compensate for that, we provide

an additional image here that represents a single confocal section through the polyp's body column and emerging bud. It corresponds to image C in Suppl. Fig. 2. DNA of all cells is stained with TOPRO (blue). GFP-positive NCPs are in green. We hope that this image supports our explanations and demonstrates that NCPs accumulate in the ectoderm of an emerging bud.

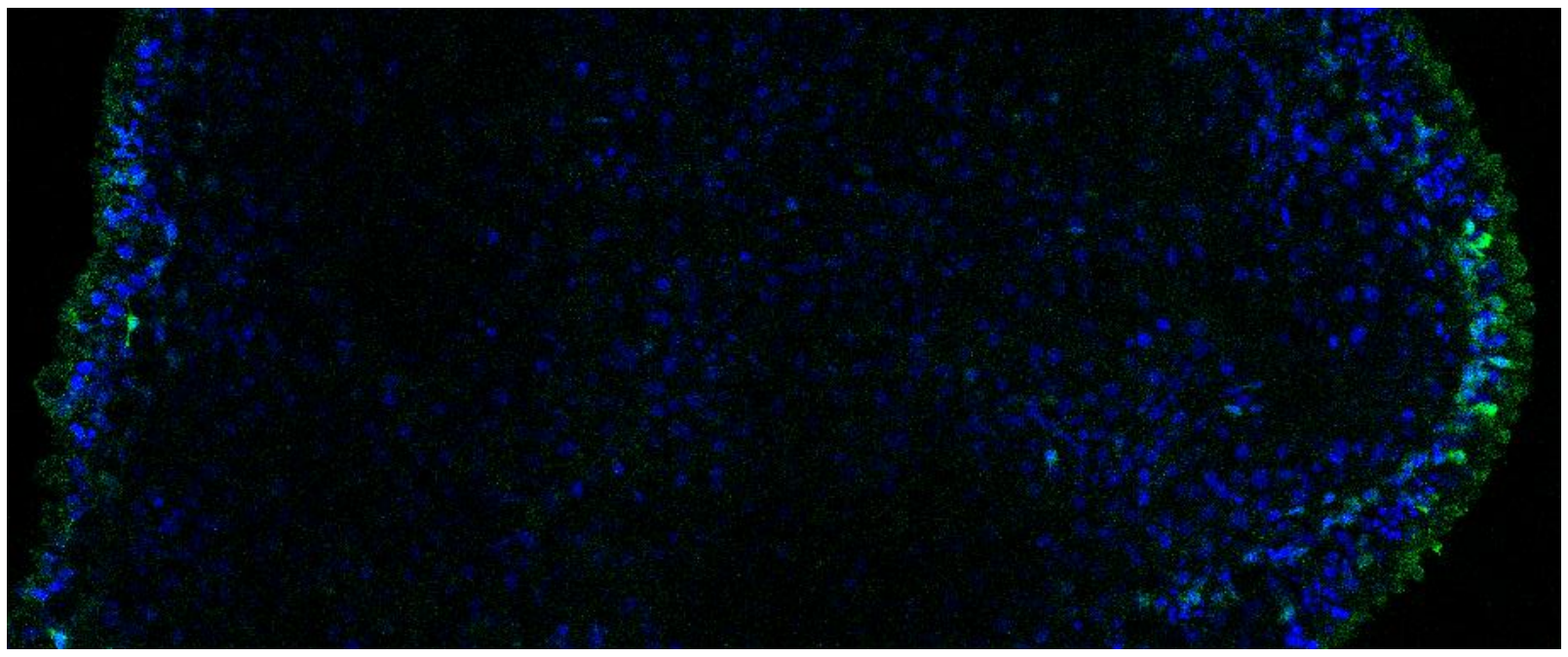

We hope this clarification adequately addresses the Reviewer's concerns.

4.line 171ff: Here some more context of how separate/interconnected the different circuits are would help. My (simplifying) instinct wants to make me believe that the circuits are separate, and responsible for different behaviors. This is obviously not so, but the basics of how N4 is integrated with N6/7 in feeding behavior should be briefly summarized. From what is described here, I assume that N4 is also responsible for mout opening in mature polyps? Is that a bit counterintuitive, given that N6/7 are located right at the mouth?

We thank you for this helpful comment. We agree that the original phrasing may have implied that these neuronal populations function as separate circuits. In *Hydra*, however, these networks are functionally interconnected. We earlier demonstrated (Giez et al., CurrBiol 2023) that N6 neurons (and also the tentacular N7, possibly associated with mechanosensation during prey movement) near the mouth are associated with sensory or stimulus-evoked responses, while the N4 network contributes to the coordination of the feeding reflex (specifically controls the mouth opening width). We have clarified this point in the revised manuscript (lines: 205-207) by mentioning how multiple networks interact to mediate mouth opening during feeding behavior and by highlighting the role of N4.

Giez et al. (2023) showed the sequence of activation of the two neuronal populations (N6 and N4) during GSH administration *in vivo* using calcium imaging. N6 neurons were activated first (more specifically, N6 sensory and then N6 ganglion neurons), followed by N4 activation to initiate mouth opening, indicating a role for N6 (particularly N6 sensory neurons) in sensory-related functions. However, ablation of either of these neuronal populations, N6 or N4, using the NTR-MTZ system alone could not completely inhibit the mouth-opening response; rather, it impaired mouth-opening durations and response times, indicating the requirement of both neuronal populations for consistent mouth opening during the feeding reflex. This highlighted that the N4 is also an integral part of the neuronal circuits that control mouth opening during feeding behavior. It is important to remember that the parent-dependent mouth opening in the bud mediated via the N4 neuronal networks is primarily initiated when the parental head is stimulated by GSH.

Therefore, N4 is once again not executing a direct stimulus-evoked response in the bud but rather coordinating the response from the parental head, where N6 sensory and ganglion cells are processing the direct GSH stimulus first. We would also like to add and highlight to the Reviewer that the N4 network is involved in engulfment and spit-out functions, as reported by Geiz *et. al*. Cell Reports, 2024, which might give a biological meaning to our observations of N4 in the bud initiating the bud's mouth opening (though in a parent-dependent manner). We hope this clarifies your question about how two different neuronal populations with defined roles (or subfunctions) can still come together to generate the same function.

5.line 198: is it known why MTZ kills only N4 neurons?

We are really sorry for this confusion. We missed out on mentioning the NTR-MTZ system used for ablating N4-specific cells, as has been previously used in our studies at Giez *et al.* 2023; Giez *et al.* 2024. The NTR-MTZ system has been developed to selectively eliminate specific neuronal populations by inducing massive DNA damage in those cells. Transgenic cells (N4 neurons in this case) constitutively produce the nitroreductase enzyme. Upon addition of the MTZ pro-drug, the cells convert it into cytotoxic nitroso-intermediates that cause double-strand DNA breaks and apoptosis in affected cells. This mechanism has been described earlier by Curado *et. al.* Nat Protoc., 2008. As we demonstrated earlier (Giez et al., 2023; Suppl Fig. 4), Mtz treatment indeed induces apoptosis in NTR-expressing neurons, as evidenced by conspicuous nucleus fragmentation in those transgenic cells prior to their disappearance. In the revised text, we have now included the genotype of the transgenic line used for this method between lines:234-239, which highlights how N4 is specifically targeted and ablated.

6.line 218ff: I come back to the different roles of N4 vs N6 in feeding behavior/mouth opening. My confusion: I assume that "mouth opening" is caused by a specific set of muscles (a ring/sphincter-like array surrounding the mouth?). Do the reported findings mean that N4 and N6 both overlap in this muscle subpopulation? Or do N4 and N6 innervate different muscles, but contraction of either of them cause the same movement (i.e., mouth opening)? This is somehow not plausible.

We thank you for this insightful comment, which highlights an important aspect of cnidarian biology that benefits from clarification. In *Hydra*, there are no discrete or specialized muscle tissues as found in vertebrates. Instead, contractile function is embedded within multifunctional epitheliomuscular cells (West, Tissue Cell, 1978). Consequently, the assumption of a sphincter-like muscle surrounding the mouth is not consistent with current anatomical and biomechanical understanding. Rather, mouth opening in *Hydra* appears to result from coordinated, distributed contractions of epitheliomuscular cells across the hypostome, rather than from a dedicated muscle ring (Carter *et. al.,* Biophys J, 2016). Recent work suggests that the closed epithelial sheet at the hypostome is mechanically opened through coordinated contractile forces and subsequently relaxes back to a closed state after feeding (Goel *et al*., Proc. R. Soc. B., 2024). In this context, neuronal subtypes such as N4 and N6 are not required to converge onto a single muscle population. Instead, they likely innervate partially distinct but mechanically coupled regions of the epitheliomuscular network. Due to the integrated

nature of the tissue, activation of different neuronal populations (N6 or N4 in this case) can give rise to the same emergent behavior (i.e., mouth opening) through coordinated tissue-level dynamics. This type of organization is consistent with the diffuse nerve net architecture of cnidarians, where behavior emerges from the interplay between network activity and tissue mechanics rather than from a one-to-one neuromuscular mapping (Dupre & Yuste, 2017; Badhiwala et al., 2021; Szymanski & Yuste, 2019; Grimmelikhuijzen & Westfall, 1995). In our previous work (Giez, CurrBiol, 2023), we investigated exactly the sequence of activations of the different cell types involved in the mouth opening response using calcium imaging: N6 sensory neurons > N6 ganglion neurons and ectodermal epithelial cells> N4 ganglion neurons (at the base of the tentacles) > endodermal epithelial cells (see Fig 5I, Giez, CurrBiol, 2023). In line with this clarification and consistent with our response to comment #4, we have revised the manuscript (lines 246–249) to more clearly indicate the role of N6 neurons as part of a sensory detection circuit within this interconnected network. We think this addresses the core confusion.

7.line 274 (related to #5): I see now that MTZ is only part of a more involved method to ablate specific cell populations. It would help to very briefly point out the mechanism.

Thank you for your attention to this again. We have clarified this in the text accordingly. Please see our response to your comment #5.

Reviewer #2 (Remarks to the Author):

Asexual budding or colony building is a central feature of many Cnidarian clades. In such a fashion a fully build animal has the capacity to develop its clone. The Hydrozoan Hydra vulgaris has been widely used for regeneration studies, but also for its capacity to propagate clonally.
In view of tissue generation during clonal reproduction a key question is how specific cell types emerge and how they are integrated into functioning tissues. This question is is particularity intriguing in the case of the nervous system.
The current study follows the emergence of several previously identified neuron types, which are critical for feeding.
They authors show that neural progenitors precede the emergence of neurons; furthermore they observe how the different types emerge and record their activity during the process.

This is overall an timely manuscript, all experiments of high quality. The study focusses on a thorough analysis mostly on cell types required in feeding.
I do not have any major concerns and from what I can see the conclusions draw are in line of the evidence presented.

One point that might be worth considering is that while the early diversification covers on neural progenitors, most/all of the subsequent analysis of neural maturation is focused on a couple subtypes. This in turn does not include all neurons and generalisations form the observation should be made carefully. I why the authors did not look into the pan-neuronal line, which for hydra exits?

Thank you very much for your very positive comments. We also want to thank you for the valuable caution regarding potential overgeneralizations. We have tweaked line:371 from “full behavioral repertoire” to just “behavioral repertoire” to tone down our conclusions.

We further agree that a pan-neuronal line could, in principle, be an attractive approach to visualize all neuronal populations. However, we would like to emphasize the main focus of our study, which is to connect circuit formation with function. To this end, we used two complementary approaches: calcium imaging with GCaMP6s reporters and chemical ablation using the NTR-MTZ system, both driven by neuron-specific promoters. Most of these lines have been validated and published earlier (Giez *et al*, 2023, Giez *et al*, 2024, Noack *et al*, 2025). Using these tools, we performed a detailed maturation analysis of defined neuronal subtypes associated with well-characterized behaviors such as feeding and spontaneous contractions. Expanding this analysis to encompass all neuronal populations and behaviors was beyond the scope of the present study. Moreover, while a pan-neuronal GCaMP line would in principle provide broader coverage, in practice it would introduce substantial analytical challenges. In particular, robustly segmenting and tracking individual neuronal populations across developmental stages—especially within the densely populated and dynamically changing head region of the forming bud—would be computationally demanding and potentially less reliable within the framework of our current analysis. In accordance with this, lines: 378-380 have been added to highlight why the specific populations of neurons were used exactly. We hope these slight modifications will help the readers realize our reasoning for using only a few populations.

Another minor point might be that Calcium imaging is only the last step in the process of wiring up of circuits ensembles, thus the developmental processes how this occurs and if this process is similar to what occurs during development can not be drawn.

We thank the Reviewer for raising this important point. We agree that calcium imaging represents a late functional readout and does not capture the developmental processes underlying circuit assembly. However, it has been earlier demonstrated (Dupre *et al*, CurrBiol, 2017) that an increase in intracellular Ca^{2+} concentration reported by the GCaMP fluorescence is a reliable proxy for neuronal activity, i.e., action potential formation in *Hydra*. Though this method has limitations, discussed elsewhere, no alternative tools, such as voltage sensors, are available to date in *Hydra*. Future work combining lineage tracing or longitudinal approaches with functional imaging could aid in addressing how these circuits are established and whether they recapitulate developmental mechanisms.

Reviewer #3 (Remarks to the Author):

The study by Sakib et al is very exciting, highly novel and thoroughly performed. It will definitely be interesting to the evolutionary neurobiology, evo-devo, and zoology fields. This manuscripts dissects mechanisms of formation of neural circuits and behaviours under their control in an animal with a very simple nervous system, freshwater polyp Hydra, during its reproduction through asexual budding. Surprisingly, the main principles turned out to be the same as in animals with much more complex nervous systems (e.g. vertebrates) suggesting that these mechanisms are very ancient and highly conserved across the animal evolution.
The experimental procedures are described in detail.

Many thanks for your positive comments.

I only have a couple of minor comments:

1. Line 89: When first introducing the new myc3::GFP//act::RFP line, it would be nice to mention what the purpose of act::RFP transgene is, not only myc3::GFP.

By introducing the second reporter, the RFP, we followed the earlier established practice (Klimovich *et al.*, Nature Protocols, 2019): RFP expressed under a ubiquitous promoter marks the entire lineage that contains the transgene, facilitates the screening of transgenic animals, and allows tracing the differentiation of stem cells and progenitors into mature derivatives (Klimovich et al., PNAS 2020). However, the actin promoter that drives RFP expression is in fact active not only in mature neurons; it also effectively stimulates RFP expression in nematocytes, though at a lower level. Therefore, RFP expression in the myc3::GFP//act::RFP is not exclusively neuronal, and RFP has not been used further in our study as a neuronal marker.

2. Line 196: It would be helpful to mention the genotype of the N4 ablation line, otherwise it is unclear why only N4 population disintegrates by the MTZ treatment.

Thank you very much for pointing this out. We have absolutely missed out on this crucial information here. The NTR enzyme is constitutively expressed under population-specific promoters. The elimination of neurons is selective since only the neurons that express NTR convert it into non-diffusible intermediates that induce DNA damage and apoptosis in transgenic cells. Neighboring non-transgenic cells are not affected.

We have now added, on lines:235-236, the genotype information for this line. We have further added "The effectiveness of this method on the same lines has been demonstrated earlier." (lines:238-239) to refer to the validated and published work on this transgenic line.

3. Line 689: It would be good to connect the genotypes to the lines described in the text (promoter t12588 is not mentioned in the text)

You are right. Mentioning the promoter information would be quite convenient here for the readers. We have now added this on lines: 301-302, "To analyse the activity of the emerging N1 network, we performed calcium imaging using a transgenic line that expresses GCaMP6s under an N1-specific promoter (HVAEP_G016171, see *transgeneis..*)" and also on line: 323.

4. *Line 944: "corresponding GFP-positive" – should be "corresponding to GFP-positive"*

Thank you for raising this point. We have added this in the text.

5. Line 945: red arrowhead seems to be missing at the figure.

Thank you very much for this hint. We have now updated the figure with this correction. However, we have changed a red arrowhead to an empty arrowhead with a white outline for better clarity, as seen below:

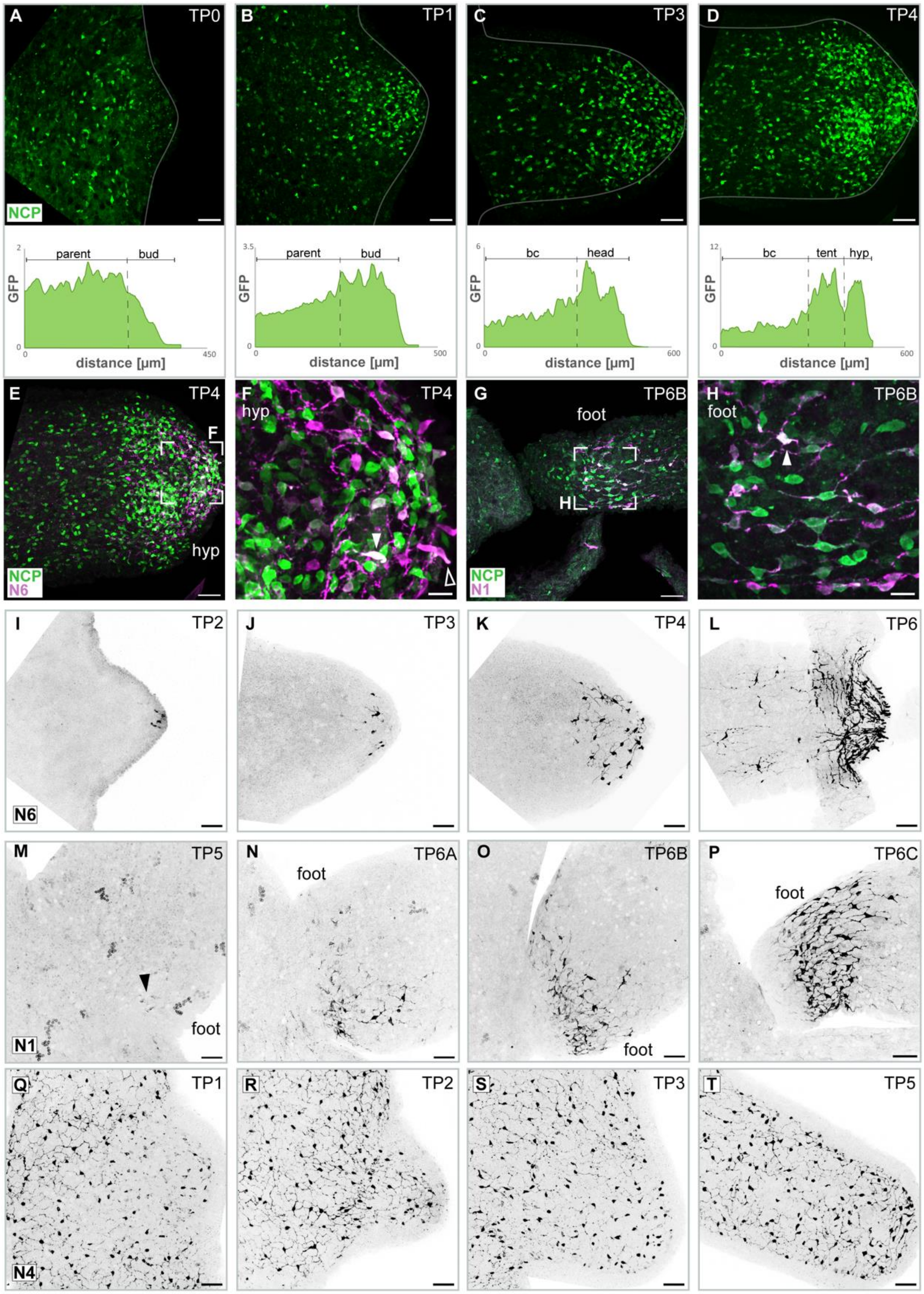
A
TP0
NCP
B
TP1
C
TP3
D
TP4
parent
bud
GFP
distance [µm]
bc
head
tent
hyp
E
TP4
F
hyp
NCP
N6
G
TP6B
foot
H
N1
I
TP2
N6
J
K
L
TP6
M
TP5
N1
N
TP6A
O
P
TP6C
Q
N4
R
S
T

The figure legend has also been updated accordingly (line: 1056)

I think this manuscript is suitable for publication.
Many thanks!

Manuscript # COMMSBIO-26-2162

Specific changes in the text and figures and comments to the Referees:

Reviewer #1 (Remarks to the Author)

This paper documents and experimentally analysis the emergence of the different neuronal circuits in the budding Hydra polyp. The paper is a masterpiece in regard to designing the experiments, visualizing the data, explaining the results. It offeres a wealth of new insights into circuit assembly.

We thank the referee for the positive and encouraging comments.

My comments for textual improvements:

1.Line 105-115: The use of specific markers for neuron subpopulations should be explained in more detail. From what I understand, Rfamide is expressed in N1, N6, N7. By using this marker how can the authors determine which of these subpopulations they are looking at?

Thank you for raising this important point, which we obviously have not addressed very well in this section. We confirm that RFamide is expressed in multiple neuronal populations (including N1, N6, and N7) and is therefore not population-specific on its own. In our study, the identity of specific neuronal populations was inferred by integrating RFamide immunostaining with spatial and temporal context during budding, as described for *Hydra* neuronal populations. The terminology and spatial location of each population of neurons have already been established (Fig 1D + Supplementary – Klimovich *et al.* PNAS 2020; Siebert *et al.*, Science, 2019), while the populations mapped on the *Hydra* polyp can be found in Fig 1D in Giez *et al.* CurrBiol, 2023, which we have also adopted in this paper in Fig 1E. In this study, the early appearance of RFamide-positive neurons at the hypostome tip is consistent with the localization of the N6 population. The tentacle-specific N7 may only appear by TP5 when tentacle buds appear on the bud's head and is identified in their localization on the young tentacles. The RFamide-positive neurons emerging later in the proximal bud region correspond to the expected position of N1 neurons. We note that we do not observe patterns consistent with N7 localization in these contexts. We have revised the manuscript (see lines:137-139 in the revised manuscript) to more clearly explain that our neuronal population assignments are based on this combined framework rather than RFamide expression alone.

2. line 122: it would help the reader if the authors explain (introduction) the composition of a circuit, like N6, of different neuron types. From l.122 I assume that N6 is composed of at least two types, sensory neurons and ganglion cells (?). Is that true for all circuits? Is it easy to distinguish the two types (morphology?). What are the implications: even though they belong to the same circuit, are these cell types molecularly distinct?

Thanks for the comment. Yes, you are absolutely right. N6 does in fact consist of two types of nerve cells based on morphological differences – sensory and ganglion. However, this is not true for all circuits; aside from N6, only N3 and N4 are known to consist of both sensory and ganglion morphologies. The sensory neurons are mainly distinguished from the ganglion cells based on the projection of their sensory cilia on the epithelium surface.

However, no molecular distinction has yet been reported between the two cell morphologies in cells of any particular neuronal population in *Hydra*. We have added a more detailed background about these cell types in the revised introduction section now (see lines 74-90).

3.line 138-150: the morphogenesis of ectoderm vs endoderm and the mouth in the bud should be explained. At the stages shown in Fig.2, does the endoderm forms a solid mass of cells in the interior of the bud? And the NPCs forming N4 vs N1/N6 form part of the endoderm vs ectoderm? Is it correct that in the parental polyp, the NPCs that will contribute to all of the NPCs entering the bud are considered ectodermal? If so, does that mean that during the early budding process they lose their ectodermal affiliation (because they give rise to ectodermal and endodermal neuron circuits)? How does the enderm become an epithelium, and how does the mouth opening form in the budding polyp?

We thank the reviewer for raising this important point regarding bud morphogenesis and the relationship between ectodermal and endodermal tissues, as well as the origin and fate of neuronal progenitor cells (NCPs).

Bud morphogenesis in *Hydra* has been described in detail in previous studies (Holstein *et al.*, 1991; Otto and Campbell, 1977). During this process, although a pronounced thickening of the endoderm is consistently observed beneath the evaginating ectodermal bud primordium (e.g., Webster and Hamilton, 1972), the endoderm does not form a solid mass of cells at any stage. Instead, both ectoderm and endoderm remain as continuous epithelial layers throughout budding. Importantly, the endoderm of the developing bud is directly continuous with that of the parental polyp, and both share a common gastric cavity (see lines 59–61 in revised manuscript). We agree that this continuity is functionally significant. It provides a structural basis for the extension of the N4 neuronal network from the parental polyp into the growing bud (Fig. 2Q–T), thereby enabling coordination between the hypostomes of the parent and the bud (Fig. 3A–D).

Regarding the origin and fate of NCPs, these cells arise in the ectoderm of the parental polyp from interstitial stem cells (Holstein and David, 1986). They give rise to all ectodermal neuronal subtypes (N1, N2, N3, N6, and N7). In addition, a subset of transient progenitors migrates through the mesoglea into the endoderm, where they differentiate into endodermal neuronal populations (N4 and N5), as well as gland cells (see lines:71-73 in the revised manuscript). The lineage relationships and differentiation trajectories of these progenitors have been described in detail by Siebert *et al.* (2019).

Thus, while all NCPs originate from the ectodermal lineage, their ability to migrate and differentiate into endodermal derivatives reflects lineage plasticity rather than a loss of origin. Both ectodermal NCPs and migrating progenitors share common molecular signatures, including expression of myc3 and myb. We leveraged this feature to generate our transgenic reporter line (Fig. S4), in which GFP expression is driven by the myc3 promoter. Consistent with the endogenous expression pattern of myc3 (Siebert et al., 2019; Lechable et al., 2023), this reporter labels the full population of immature progenitors irrespective of their final localization.

We assume that the question of the Reviewer arises mainly due to the fact that most of the images we present are Maximum Intensity Projections from confocal Z-stacks, which do not allow for perceiving the depth of cells' localization. To compensate for that, we provide

an additional image here that represents a single confocal section through the polyp's body column and emerging bud. It corresponds to image C in Suppl. Fig. 2. DNA of all cells is stained with TOPRO (blue). GFP-positive NCPs are in green. We hope that this image supports our explanations and demonstrates that NCPs accumulate in the ectoderm of an emerging bud.

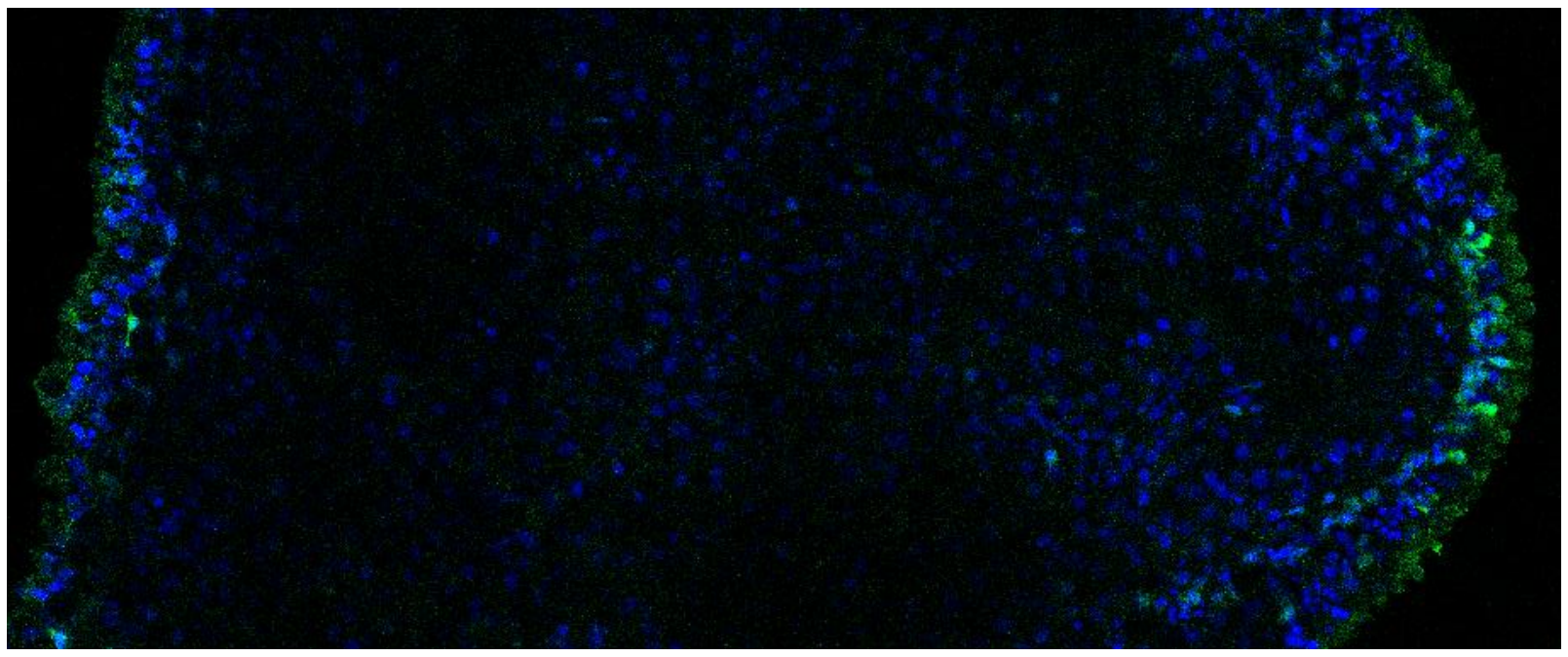

We hope this clarification adequately addresses the Reviewer's concerns.

4.line 171ff: Here some more context of how separate/interconnected the different circuits are would help. My (simplifying) instinct wants to make me believe that the circuits are separate, and responsible for different behaviors. This is obviously not so, but the basics of how N4 is integrated with N6/7 in feeding behavior should be briefly summarized. From what is described here, I assume that N4 is also responsible for mout opening in mature polyps? Is that a bit counterintuitive, given that N6/7 are located right at the mouth?

We thank you for this helpful comment. We agree that the original phrasing may have implied that these neuronal populations function as separate circuits. In *Hydra*, however, these networks are functionally interconnected. We earlier demonstrated (Giez et al., CurrBiol 2023) that N6 neurons (and also the tentacular N7, possibly associated with mechanosensation during prey movement) near the mouth are associated with sensory or stimulus-evoked responses, while the N4 network contributes to the coordination of the feeding reflex (specifically controls the mouth opening width). We have clarified this point in the revised manuscript (lines: 205-207) by mentioning how multiple networks interact to mediate mouth opening during feeding behavior and by highlighting the role of N4.

Giez et al. (2023) showed the sequence of activation of the two neuronal populations (N6 and N4) during GSH administration *in vivo* using calcium imaging. N6 neurons were activated first (more specifically, N6 sensory and then N6 ganglion neurons), followed by N4 activation to initiate mouth opening, indicating a role for N6 (particularly N6 sensory neurons) in sensory-related functions. However, ablation of either of these neuronal populations, N6 or N4, using the NTR-MTZ system alone could not completely inhibit the mouth-opening response; rather, it impaired mouth-opening durations and response times, indicating the requirement of both neuronal populations for consistent mouth opening during the feeding reflex. This highlighted that the N4 is also an integral part of the neuronal circuits that control mouth opening during feeding behavior. It is important to remember that the parent-dependent mouth opening in the bud mediated via the N4 neuronal networks is primarily initiated when the parental head is stimulated by GSH.

Therefore, N4 is once again not executing a direct stimulus-evoked response in the bud but rather coordinating the response from the parental head, where N6 sensory and ganglion cells are processing the direct GSH stimulus first. We would also like to add and highlight to the Reviewer that the N4 network is involved in engulfment and spit-out functions, as reported by Geiz *et. al*. Cell Reports, 2024, which might give a biological meaning to our observations of N4 in the bud initiating the bud's mouth opening (though in a parent-dependent manner). We hope this clarifies your question about how two different neuronal populations with defined roles (or subfunctions) can still come together to generate the same function.

5.line 198: is it known why MTZ kills only N4 neurons?

We are really sorry for this confusion. We missed out on mentioning the NTR-MTZ system used for ablating N4-specific cells, as has been previously used in our studies at Giez *et al.* 2023; Giez *et al.* 2024. The NTR-MTZ system has been developed to selectively eliminate specific neuronal populations by inducing massive DNA damage in those cells. Transgenic cells (N4 neurons in this case) constitutively produce the nitroreductase enzyme. Upon addition of the MTZ pro-drug, the cells convert it into cytotoxic nitroso-intermediates that cause double-strand DNA breaks and apoptosis in affected cells. This mechanism has been described earlier by Curado *et. al.* Nat Protoc., 2008. As we demonstrated earlier (Giez et al., 2023; Suppl Fig. 4), Mtz treatment indeed induces apoptosis in NTR-expressing neurons, as evidenced by conspicuous nucleus fragmentation in those transgenic cells prior to their disappearance. In the revised text, we have now included the genotype of the transgenic line used for this method between lines:234-239, which highlights how N4 is specifically targeted and ablated.

6.line 218ff: I come back to the different roles of N4 vs N6 in feeding behavior/mouth opening. My confusion: I assume that "mouth opening" is caused by a specific set of muscles (a ring/sphincter-like array surrounding the mouth?). Do the reported findings mean that N4 and N6 both overlap in this muscle subpopulation? Or do N4 and N6 innervate different muscles, but contraction of either of them cause the same movement (i.e., mouth opening)? This is somehow not plausible.

We thank you for this insightful comment, which highlights an important aspect of cnidarian biology that benefits from clarification. In *Hydra*, there are no discrete or specialized muscle tissues as found in vertebrates. Instead, contractile function is embedded within multifunctional epitheliomuscular cells (West, Tissue Cell, 1978). Consequently, the assumption of a sphincter-like muscle surrounding the mouth is not consistent with current anatomical and biomechanical understanding. Rather, mouth opening in *Hydra* appears to result from coordinated, distributed contractions of epitheliomuscular cells across the hypostome, rather than from a dedicated muscle ring (Carter *et. al.,* Biophys J, 2016). Recent work suggests that the closed epithelial sheet at the hypostome is mechanically opened through coordinated contractile forces and subsequently relaxes back to a closed state after feeding (Goel *et al*., Proc. R. Soc. B., 2024). In this context, neuronal subtypes such as N4 and N6 are not required to converge onto a single muscle population. Instead, they likely innervate partially distinct but mechanically coupled regions of the epitheliomuscular network. Due to the integrated

nature of the tissue, activation of different neuronal populations (N6 or N4 in this case) can give rise to the same emergent behavior (i.e., mouth opening) through coordinated tissue-level dynamics. This type of organization is consistent with the diffuse nerve net architecture of cnidarians, where behavior emerges from the interplay between network activity and tissue mechanics rather than from a one-to-one neuromuscular mapping (Dupre & Yuste, 2017; Badhiwala et al., 2021; Szymanski & Yuste, 2019; Grimmelikhuijzen & Westfall, 1995). In our previous work (Giez, CurrBiol, 2023), we investigated exactly the sequence of activations of the different cell types involved in the mouth opening response using calcium imaging: N6 sensory neurons > N6 ganglion neurons and ectodermal epithelial cells> N4 ganglion neurons (at the base of the tentacles) > endodermal epithelial cells (see Fig 5I, Giez, CurrBiol, 2023). In line with this clarification and consistent with our response to comment #4, we have revised the manuscript (lines 246–249) to more clearly indicate the role of N6 neurons as part of a sensory detection circuit within this interconnected network. We think this addresses the core confusion.

7.line 274 (related to #5): I see now that MTZ is only part of a more involved method to ablate specific cell populations. It would help to very briefly point out the mechanism.

Thank you for your attention to this again. We have clarified this in the text accordingly. Please see our response to your comment #5.

Reviewer #2 (Remarks to the Author):

Asexual budding or colony building is a central feature of many Cnidarian clades. In such a fashion a fully build animal has the capacity to develop its clone. The Hydrozoan Hydra vulgaris has been widely used for regeneration studies, but also for its capacity to propagate clonally.
In view of tissue generation during clonal reproduction a key question is how specific cell types emerge and how they are integrated into functioning tissues. This question is is particularity intriguing in the case of the nervous system.
The current study follows the emergence of several previously identified neuron types, which are critical for feeding.
They authors show that neural progenitors precede the emergence of neurons; furthermore they observe how the different types emerge and record their activity during the process.

This is overall an timely manuscript, all experiments of high quality. The study focusses on a thorough analysis mostly on cell types required in feeding.
I do not have any major concerns and from what I can see the conclusions draw are in line of the evidence presented.

One point that might be worth considering is that while the early diversification covers on neural progenitors, most/all of the subsequent analysis of neural maturation is focused on a couple subtypes. This in turn does not include all neurons and generalisations form the observation should be made carefully. I why the authors did not look into the pan-neuronal line, which for hydra exits?

Thank you very much for your very positive comments. We also want to thank you for the valuable caution regarding potential overgeneralizations. We have tweaked line:371 from “full behavioral repertoire” to just “behavioral repertoire” to tone down our conclusions.

We further agree that a pan-neuronal line could, in principle, be an attractive approach to visualize all neuronal populations. However, we would like to emphasize the main focus of our study, which is to connect circuit formation with function. To this end, we used two complementary approaches: calcium imaging with GCaMP6s reporters and chemical ablation using the NTR-MTZ system, both driven by neuron-specific promoters. Most of these lines have been validated and published earlier (Giez *et al*, 2023, Giez *et al*, 2024, Noack *et al*, 2025). Using these tools, we performed a detailed maturation analysis of defined neuronal subtypes associated with well-characterized behaviors such as feeding and spontaneous contractions. Expanding this analysis to encompass all neuronal populations and behaviors was beyond the scope of the present study. Moreover, while a pan-neuronal GCaMP line would in principle provide broader coverage, in practice it would introduce substantial analytical challenges. In particular, robustly segmenting and tracking individual neuronal populations across developmental stages—especially within the densely populated and dynamically changing head region of the forming bud—would be computationally demanding and potentially less reliable within the framework of our current analysis. In accordance with this, lines: 378-380 have been added to highlight why the specific populations of neurons were used exactly. We hope these slight modifications will help the readers realize our reasoning for using only a few populations.

Another minor point might be that Calcium imaging is only the last step in the process of wiring up of circuits ensembles, thus the developmental processes how this occurs and if this process is similar to what occurs during development can not be drawn.

We thank the Reviewer for raising this important point. We agree that calcium imaging represents a late functional readout and does not capture the developmental processes underlying circuit assembly. However, it has been earlier demonstrated (Dupre *et al*, CurrBiol, 2017) that an increase in intracellular Ca^{2+} concentration reported by the GCaMP fluorescence is a reliable proxy for neuronal activity, i.e., action potential formation in *Hydra*. Though this method has limitations, discussed elsewhere, no alternative tools, such as voltage sensors, are available to date in *Hydra*. Future work combining lineage tracing or longitudinal approaches with functional imaging could aid in addressing how these circuits are established and whether they recapitulate developmental mechanisms.

Reviewer #3 (Remarks to the Author):

The study by Sakib et al is very exciting, highly novel and thoroughly performed. It will definitely be interesting to the evolutionary neurobiology, evo-devo, and zoology fields. This manuscripts dissects mechanisms of formation of neural circuits and behaviours under their control in an animal with a very simple nervous system, freshwater polyp Hydra, during its reproduction through asexual budding. Surprisingly, the main principles turned out to be the same as in animals with much more complex nervous systems (e.g. vertebrates) suggesting that these mechanisms are very ancient and highly conserved across the animal evolution.
The experimental procedures are described in detail.

Many thanks for your positive comments.

I only have a couple of minor comments:

1. Line 89: When first introducing the new myc3::GFP//act::RFP line, it would be nice to mention what the purpose of act::RFP transgene is, not only myc3::GFP.

By introducing the second reporter, the RFP, we followed the earlier established practice (Klimovich *et al.*, Nature Protocols, 2019): RFP expressed under a ubiquitous promoter marks the entire lineage that contains the transgene, facilitates the screening of transgenic animals, and allows tracing the differentiation of stem cells and progenitors into mature derivatives (Klimovich et al., PNAS 2020). However, the actin promoter that drives RFP expression is in fact active not only in mature neurons; it also effectively stimulates RFP expression in nematocytes, though at a lower level. Therefore, RFP expression in the myc3::GFP//act::RFP is not exclusively neuronal, and RFP has not been used further in our study as a neuronal marker.

2. Line 196: It would be helpful to mention the genotype of the N4 ablation line, otherwise it is unclear why only N4 population disintegrates by the MTZ treatment.

Thank you very much for pointing this out. We have absolutely missed out on this crucial information here. The NTR enzyme is constitutively expressed under population-specific promoters. The elimination of neurons is selective since only the neurons that express NTR convert it into non-diffusible intermediates that induce DNA damage and apoptosis in transgenic cells. Neighboring non-transgenic cells are not affected.

We have now added, on lines:235-236, the genotype information for this line. We have further added "The effectiveness of this method on the same lines has been demonstrated earlier." (lines:238-239) to refer to the validated and published work on this transgenic line.

3. Line 689: It would be good to connect the genotypes to the lines described in the text (promoter t12588 is not mentioned in the text)

You are right. Mentioning the promoter information would be quite convenient here for the readers. We have now added this on lines: 301-302, "To analyse the activity of the emerging N1 network, we performed calcium imaging using a transgenic line that expresses GCaMP6s under an N1-specific promoter (HVAEP_G016171, see *transgeneis..*)" and also on line: 323.

4. *Line 944: "corresponding GFP-positive" – should be "corresponding to GFP-positive"*

Thank you for raising this point. We have added this in the text.

5. Line 945: red arrowhead seems to be missing at the figure.

Thank you very much for this hint. We have now updated the figure with this correction. However, we have changed a red arrowhead to an empty arrowhead with a white outline for better clarity, as seen below:

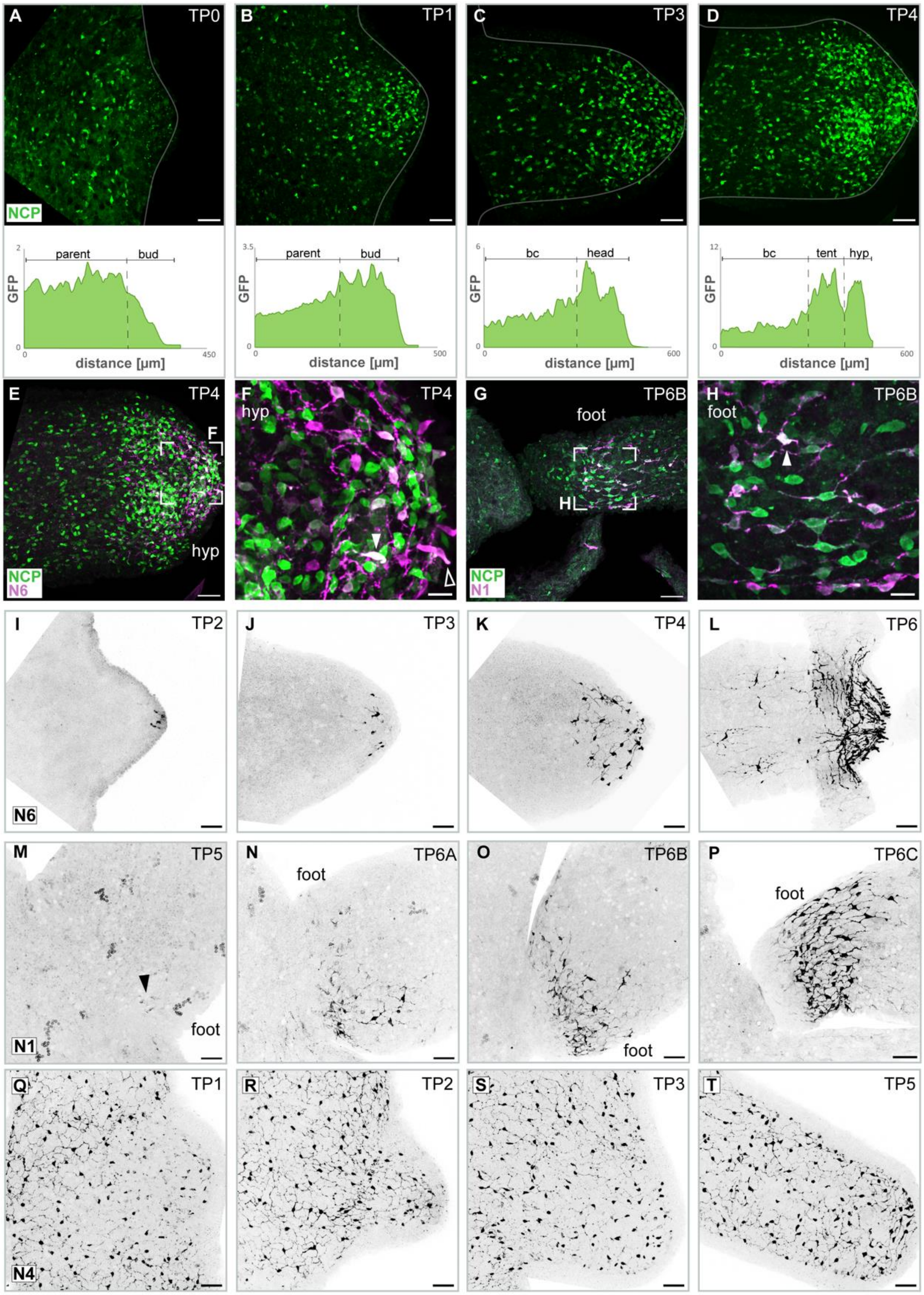

A TP0
NCP
B TP1
C TP3
D TP4
parent
bud
GFP
distance [µm]
bc
head
tent
hyp
E TP4
F
NCP
N6
F TP4
G TP6B
foot
H
NCP
N1
H TP6B
I TP2
N6
J TP3
K TP4
L TP6
M TP5
N1
N TP6A
O TP6B
P TP6C
Q TP1
N4
R TP2
S TP3
T TP5

The figure legend has also been updated accordingly (line: 1056)

I think this manuscript is suitable for publication.
Many thanks!
