# Supplementary material for: In vivo replication of a fully functional nerve net in a clonally reproducing animal
Source: Commun Biol. 2026 Oct 22;9:1389. doi: 10.1038/s42003-026-11184-x (PMC13690700; doi:10.1038/s42003-026-11184-x)
Supplement: Supplementary file 2 [file 42003_2026_11184_MOESM2_ESM.pdf]

SUPPLEMENTARY INFORMATION

In vivo replication of a fully functional nerve net in a clonally reproducing animal

Ehsan Sakib*, Lisa-Marie Hofacker*, Christopher Noack*, Finn Ströbel, Christoph Giez, Jörg Wittlieb, Alexander Klimovich, Thomas C.G. Bosch

This PDF file includes:

Supplementary Figures 1 to 7:

as Fig. S1 to S7

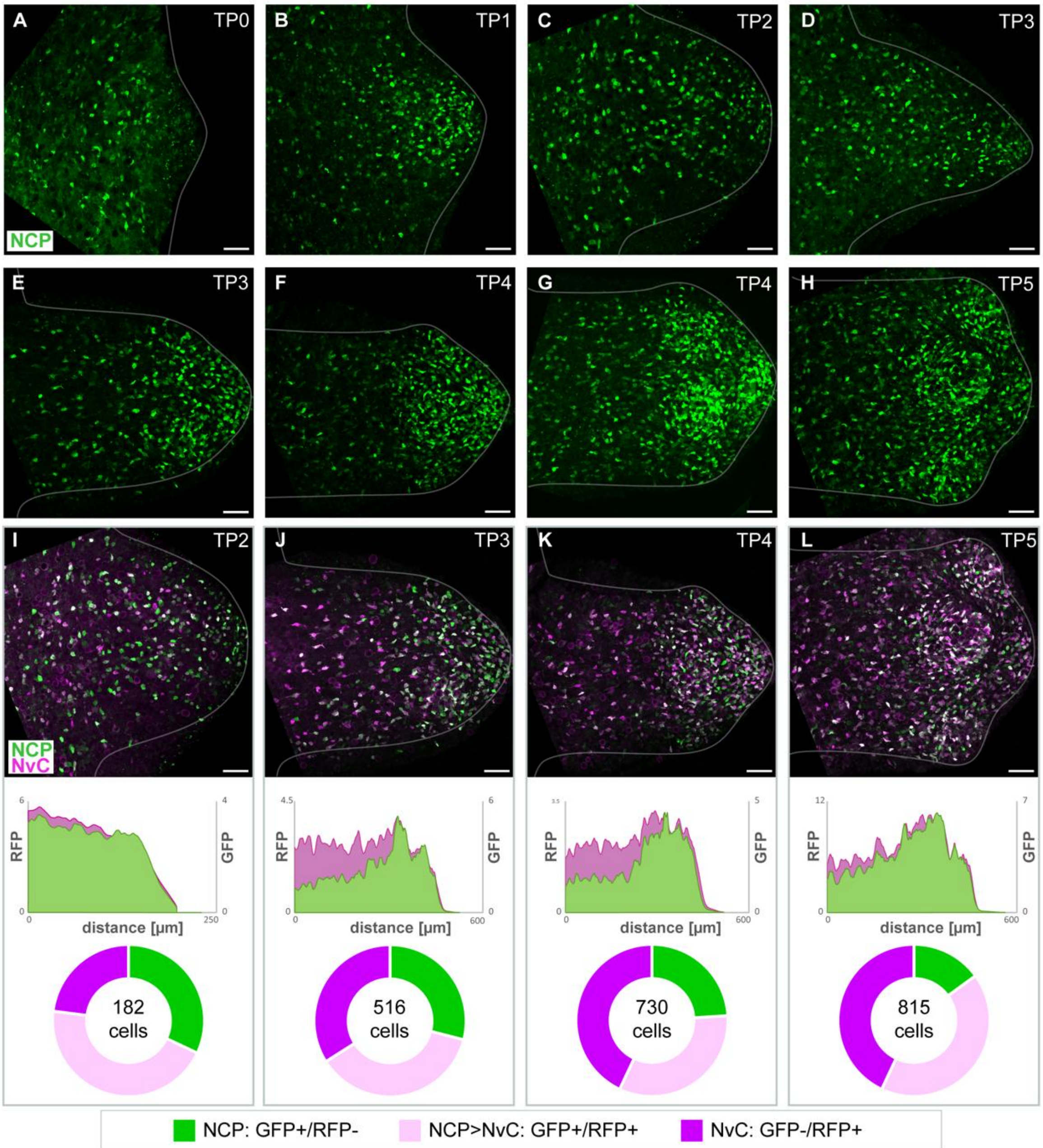

Fig. S1: Pattern formation of NCPs precedes the formation of a patterned nervous system. A-H Immunohistochemistry (representative images, scale bar 50 µm) visualizing a more detailed resolution of NCP dynamics during early bud development. (*I-L*) Immunohistochemistry (representative images, scale bar 50 µm) and corresponding GFP (green) and RFP (magenta) signal density diagrams (arbitrary unit x10^3), visualizing neuronal maturation during early bud development. Thereby, NCPs (green) differentiate into mature neurons (magenta) by passing an intermediate state (white), which is characterized by current maturation.

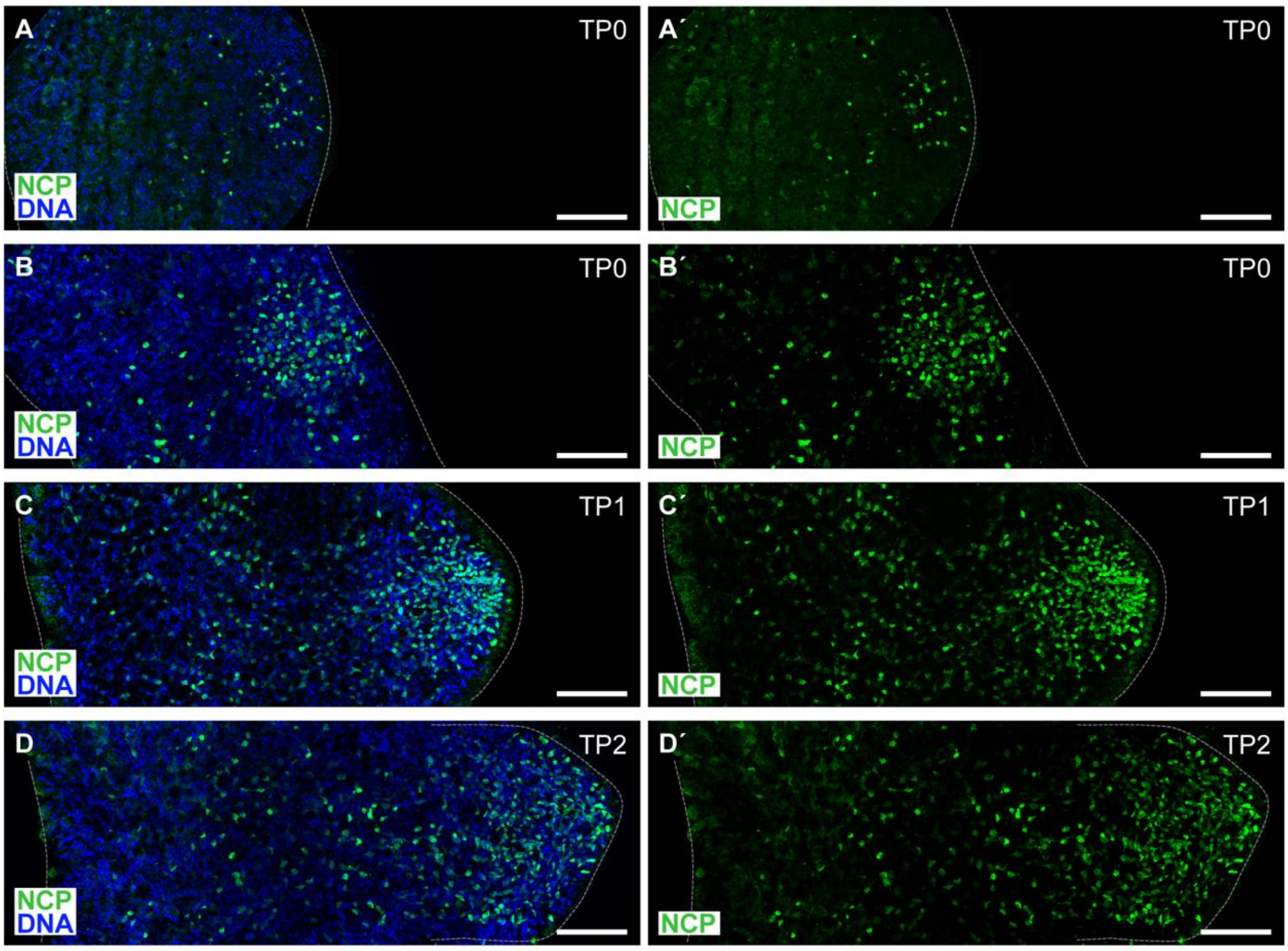

Fig. S2: NCPs accumulate in the early bud tip. A-D′ Immunohistochemistry (representative images, scale bar 100 µm) visualizing a more detailed resolution of NCP dynamics in a growing bud tip from TP0-TP2 for further replicates, as compared to **Fig.S1**. (*A-D*) (left panel) represents accumulation of GFP-positive NCPs (green) merged with DNA staining (blue), while (*A´-D´*) (right panel) shows only the GFP-positive NCPs (green) for the respective bud representative of each time point.

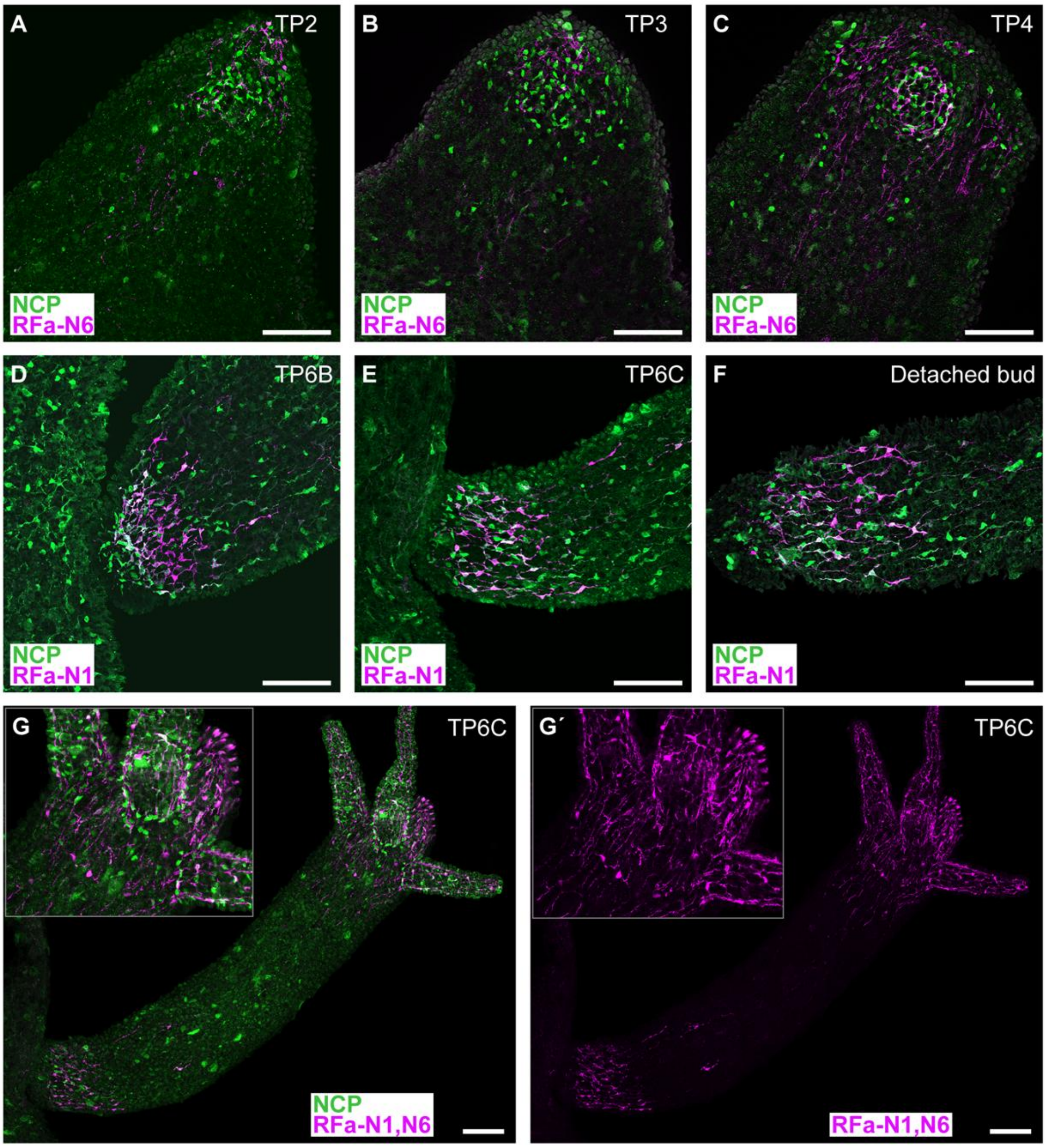

Fig. S3: Detailed overview of the accumulation of NCPs preceding specific neuronal subtype formation (*A-G'*). Immunohistochemistry (representative images, scale bar 100 µm) visualizing GFP-positive NCPs and Rfamide-positive neurons that represent N6 neurons on the distal end and N1 neurons on the proximal end of the bud. A-C (top panel) represent double-stained buds from TP2-TP4, where accumulation of NCPs (green) precedes formation of Rfamide-positive neurons (magenta) such as N6 on the hypostome. D-F (middle panel) show double-stained buds from TP6B up to a detached bud where NCP (green) and developing Rfamide-positive N1 neurons on the peduncle are densely packed. G-G′ (bottom panel) shows a whole TP6 bud, double-stained for GFP and Rfamide on G (left) and a single-channel representative (right) for only Rfamide-positive neurons. Head region (distal end) is zoomed in on the top-left of each image for a better view of the densely packed Rfamide-positive neurons by this time point of the bud.

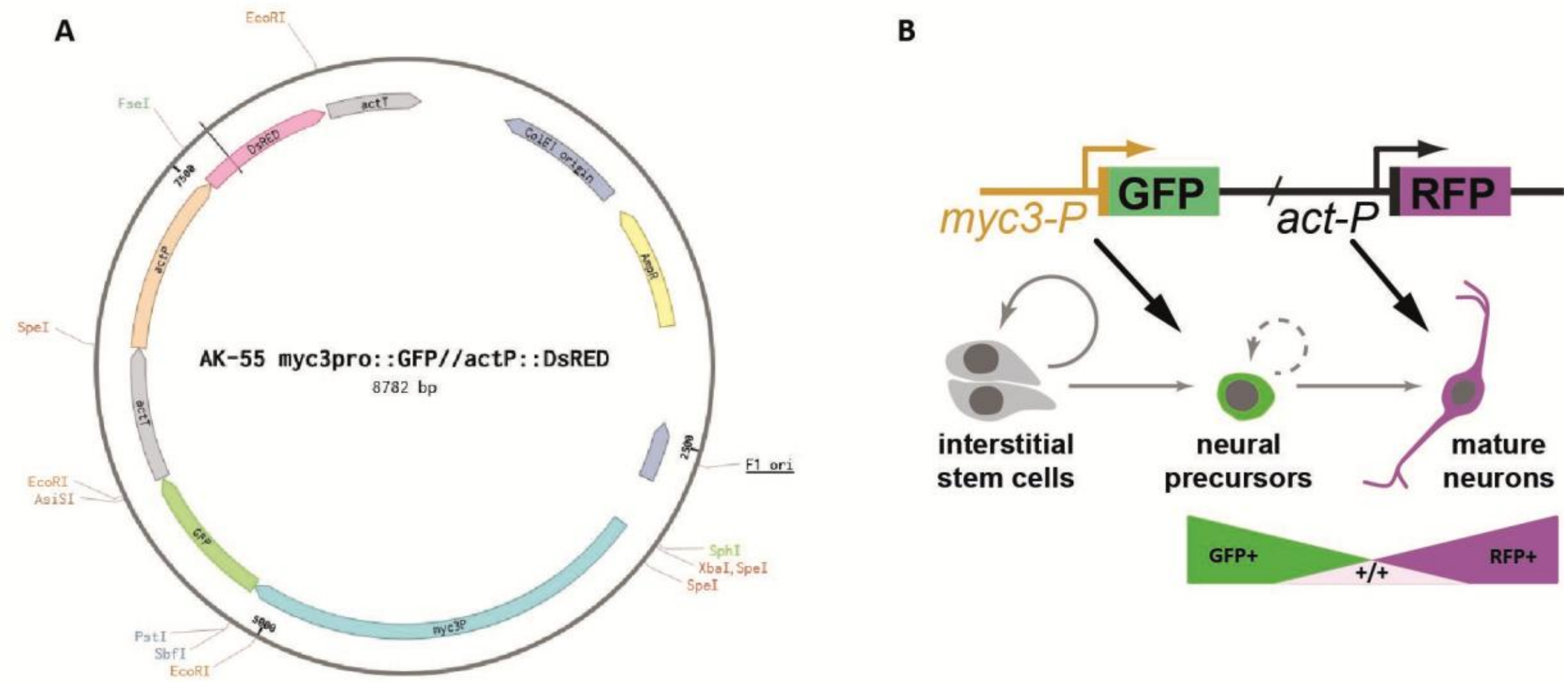

Fig. S4: A novel transgenic line enables visualization of NCPs and neuronal maturation. A Plasmid used for generating the transgenic line via embryo microinjection (see Transgenesis and constructs in Materials and methods). **B** The *myc3* promoter is active in NCPs, labelling them with GFP, while the *actin* promoter only becomes active during maturation of a neuron, labelling differentiated neurons with RFP. During maturation, a neuronal cell therefore passes a transient state in which it is labelled by both GFP and RFP.

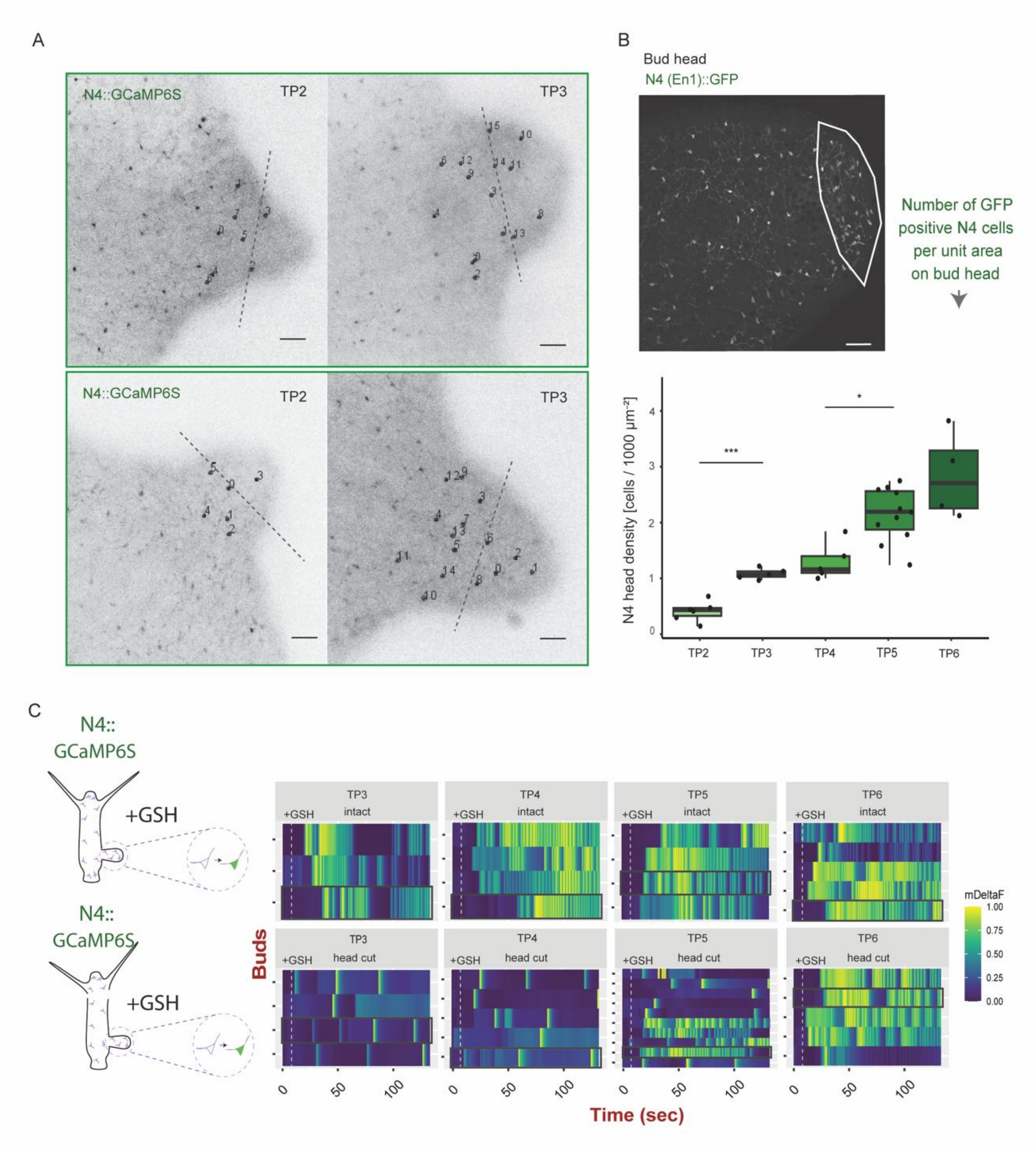

Fig. S5: Control of the mouth opening in buds through the N4 network. A Active neurons of the N4 network during a baseline event in TP2 and TP3 of the same animal show an increased density of active cells at the distal end of the bud (n=3)**. B** Anti-GFP staining showing N4 counts in the head region to score the density of the neurons, revealing a significant increase between TP2 and TP3, as well as between TP4 and TP5; Welch's ANOVA, p=0.000009109243; Games-Howell (* p<0,05; ** p<0,01; *** p<0,001). The image with GFP-labeled cells in the top panel represents the selection of the head region by drawing a polygon and counting cells in the region for the density calculations as neurons per 1000 µm^2. n=5-10. Highlighted replicate with a dark green box means this replicate was also shown before in **Fig. 3D.** Scale bar: 50 µm (*A,B*). **C** Spike train analysis comparing the influence of reduced glutathione (GSH) on the N4 population activity reveals distinct responses based on the presence or absence of the parent head across bud developmental stages. Schematic (left panel) shows the parent's

head intact or cut. Each plot represents one stage for either an intact or decapitated (head cut) budding polyp. Each row on each plot represents one individual replicate with mean fluorescence intensity for the entire N4 network for that bud. n= 3-10.

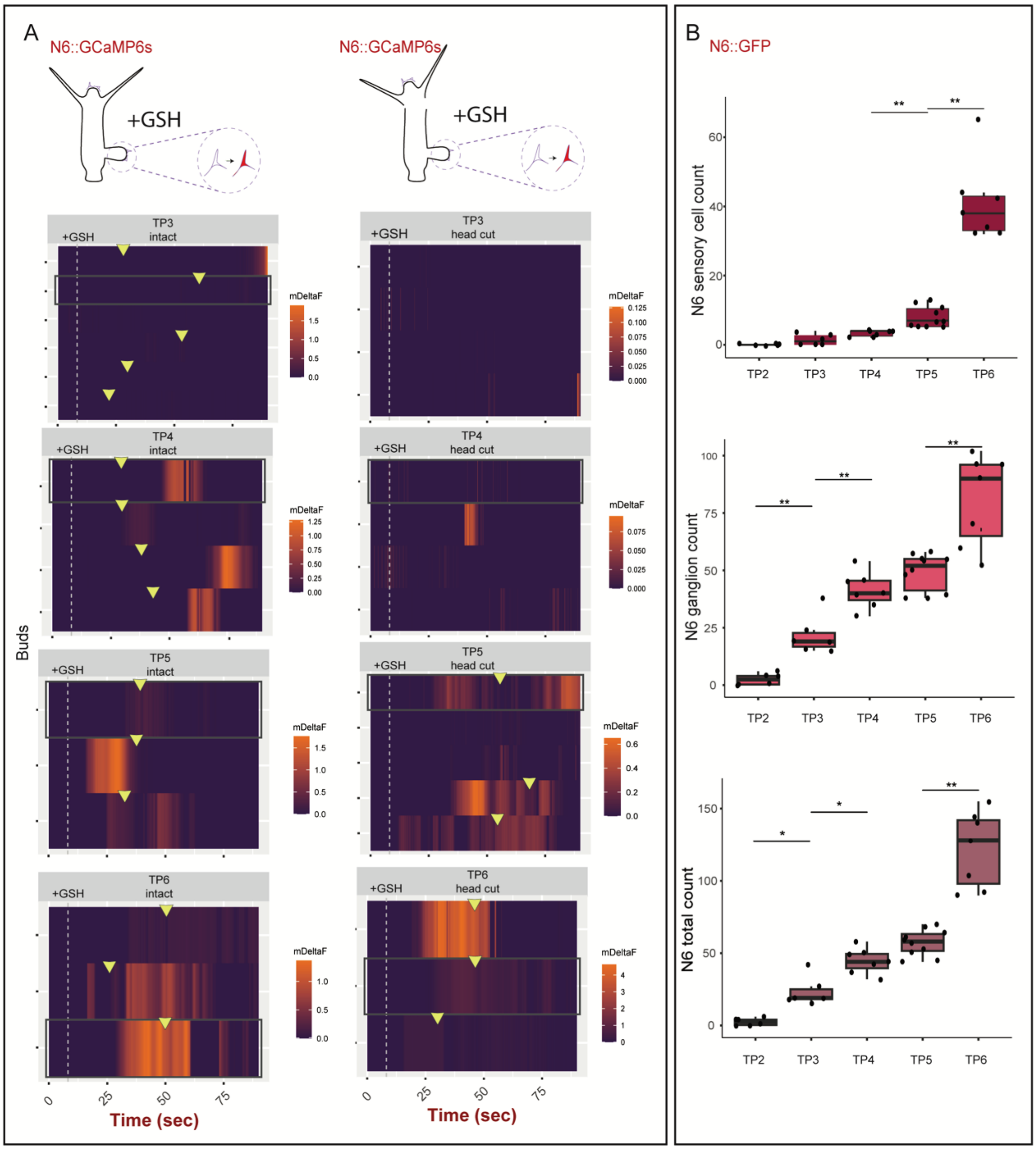

Fig. S6: Control of mouth-opening in buds through the N6 network. A Spike train analysis of N6 population in the developing bud at different time-points in response to GSH. Arrow (yellow-green) on top of rows indicates the approximate time when mouth opening initiates. Absence of an arrow on any row indicates absence of a mouth opening. The comparison between buds of intact polyps (*left panel*) and decapitated polyps (*right panel*) shows TP5 is the first stage where N6 cells can respond to the stimulus. In TP5-6, mouth opening initiates after the onset of N6 activity. Each plot represents a single bud stage. Each row on each plot represents an individual replicate (n=3-5). Highlighted replicate with a green box means this replicate was also shown before in **Fig. *3I*. B** Anti-GFP staining reveals ganglion neurons from the N6 population (top panel) first appear at TP2 on the tip of the growing head of the bud and significantly expand between TP2 and TP3, TP3 and TP4 and also between TP5 and TP6; Kruskal-Wallis, p=0.000005274; Pairwise Wilcoxon Rank Sum Test, Holm correction. Total neurons calculated

by adding sensory and ganglion numbers show high significance going from TP5 to TP6; Welch's one-way ANOVA, p=0.000000000157; Games-Howell (* p<0.05; ** p<0.01). n= 6 (TP2), 6 (TP3), 7 (TP4), 10 (TP5), and, 7 (TP6).

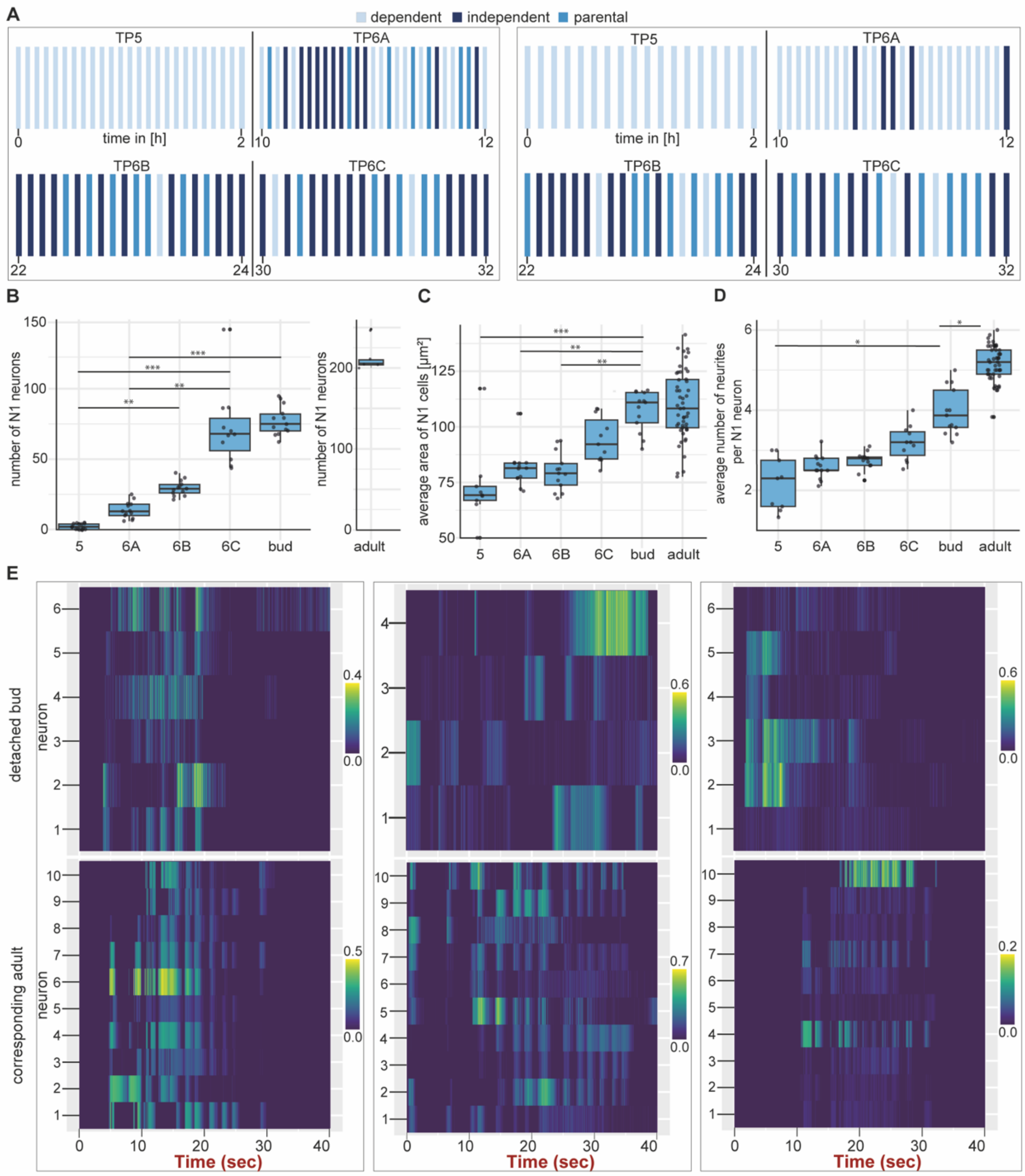

Fig. S7: A Behavioral pattern of parental polyp and bud of two animals followed throughout the progression of their buds from TP5 to TP6C. The contractile behavior was plotted in an actogram at 2-hour intervals across the late developmental stages. The different colors represent three different behaviors: DC, IC, and PC. **B** RFamide staining reveals the first N1 neurons appearing in a cluster of up to 3 neurons in TP5 buds. The number of N1 neurons increased more than two-fold with each new morphological stage until TP6C. Approximately 210 N1 neurons are present in the peduncle of adult polyps. **C** The average area of N1 neurons increased during development. Horizontal and vertical measurements were taken from individual neurons and multiplied to calculate the area. The average of 7-10 random measurements was taken for each replicate. **D** The average number of N1 neurites per neuron increased across the developmental time points. For each replicate, the number of neurites of 7-10 neurons was counted, and the average was calculated. The measurements were taken from

Immunohistochemistry Images, in which RFamide-positive cells were stained. n= 5-50, Kruskal-Wallis, Dunn Test with Bonferroni correction (* $p<0,05$; ** $p<0,01$; *** $p<0,001$). **E** Single-neuron tracking of N1 neurons in detached *Hydra* buds revealed an asynchronous spiking pattern compared to the corresponding adult, exhibiting high synchrony during contractile behavior.

Supplementary Data 1

Contains source data for all graphs in the paper.

Supplementary Video 1: Mouth opening response of a TP3 bud with intact parent head to 10 μM GSH.

Supplementary Video 2: Mouth opening response of a TP3 bud with parent head cut to 10 μM GSH.

Supplementary Video 3: Independent spontaneous contraction event of a TP6 bud.

Supplementary Video 4: Independent spontaneous contraction event of a parent with TP6 bud.
