# Supplementary material for: In vivo replication of a fully functional nerve net in a clonally reproducing animal
Source: Commun Biol. 2026 Oct 22;9:1389. doi: 10.1038/s42003-026-11184-x (PMC13690700; doi:10.1038/s42003-026-11184-x)
Supplement: Supplementary file 3 [file 42003_2026_11184_MOESM8_ESM.pdf]

nature portfolio

Corresponding author(s): Klimovich A., Bosch T.C.G.

Last updated by author(s): 30/07/2026

Reporting Summary

Nature Portfolio wishes to improve the reproducibility of the work that we publish. This form provides structure for consistency and transparency in reporting. For further information on Nature Portfolio policies, see our Editorial Policies and the Editorial Policy Checklist.

Statistics

For all statistical analyses, confirm that the following items are present in the figure legend, table legend, main text, or Methods section.

n/a	Confirmed	
☐	☒	The exact sample size (*n*) for each experimental group/condition, given as a discrete number and unit of measurement
☐	☒	A statement on whether measurements were taken from distinct samples or whether the same sample was measured repeatedly
☐	☒	The statistical test(s) used AND whether they are one- or two-sided<br>*Only common tests should be described solely by name; describe more complex techniques in the Methods section.*
☐	☒	A description of all covariates tested
☐	☒	A description of any assumptions or corrections, such as tests of normality and adjustment for multiple comparisons
☐	☒	A full description of the statistical parameters including central tendency (e.g. means) or other basic estimates (e.g. regression coefficient) AND variation (e.g. standard deviation) or associated estimates of uncertainty (e.g. confidence intervals)
☒	☐	For null hypothesis testing, the test statistic (e.g. *F*, *t*, *r*) with confidence intervals, effect sizes, degrees of freedom and *P* value noted<br>*Give P values as exact values whenever suitable.*
☒	☐	For Bayesian analysis, information on the choice of priors and Markov chain Monte Carlo settings
☒	☐	For hierarchical and complex designs, identification of the appropriate level for tests and full reporting of outcomes
☒	☐	Estimates of effect sizes (e.g. Cohen's *d*, Pearson's *r*), indicating how they were calculated

Our web collection on statistics for biologists contains articles on many of the points above.

Software and code

Policy information about availability of computer code

Data collection	ICY (v2.5.2)<br>Zeiss ZEN blue (v3.6)<br>FIJI IMAGEJ (v2.14)
Data analysis	No original codes were generated in this study. Previously published custom R codes (Giez et al, Current Biology, 2023) were used for analyzing the calcium imaging data. The codes are available upon request to the lead author at tbosch@zoologie.uni-kiel.de. Giez et al, Cell Reports, 2024 also used similar adopted codes (deposited at https://github.com/ChristophGW/Cell-Reports_Satiety-Hydra). Codes in this study were adjusted to fit data structure and end visualization only and can be provided upon request to the authors.

For manuscripts utilizing custom algorithms or software that are central to the research but not yet described in published literature, software must be made available to editors and reviewers. We strongly encourage code deposition in a community repository (e.g. GitHub). See the Nature Portfolio guidelines for submitting code & software for further information.

Data

Policy information about availability of data

All manuscripts must include a data availability statement. This statement should provide the following information, where applicable:

- Accession codes, unique identifiers, or web links for publicly available datasets
- A description of any restrictions on data availability
- For clinical datasets or third party data, please ensure that the statement adheres to our policy

All numeric data used for making bar, box or line plots are provided as Supplementary Data 1. All other maging data that support the findings of this study are available from the corresponding authors upon request (tbosch@zoologie.uni-kiel.de).

Research involving human participants, their data, or biological material

Policy information about studies with human participants or human data. See also policy information about sex, gender (identity/presentation), and sexual orientation and race, ethnicity and racism.

Reporting on sex and gender	*Use the terms sex (biological attribute) and gender (shaped by social and cultural circumstances) carefully in order to avoid confusing both terms. Indicate if findings apply to only one sex or gender; describe whether sex and gender were considered in study design; whether sex and/or gender was determined based on self-reporting or assigned and methods used. Provide in the source data disaggregated sex and gender data, where this information has been collected, and if consent has been obtained for sharing of individual-level data; provide overall numbers in this Reporting Summary. Please state if this information has not been collected. Report sex- and gender-based analyses where performed, justify reasons for lack of sex- and gender-based analysis.*
Reporting on race, ethnicity, or other socially relevant groupings	*Please specify the socially constructed or socially relevant categorization variable(s) used in your manuscript and explain why they were used. Please note that such variables should not be used as proxies for other socially constructed/relevant variables (for example, race or ethnicity should not be used as a proxy for socioeconomic status). Provide clear definitions of the relevant terms used, how they were provided (by the participants/respondents, the researchers, or third parties), and the method(s) used to classify people into the different categories (e.g. self-report, census or administrative data, social media data, etc.) Please provide details about how you controlled for confounding variables in your analyses.*
Population characteristics	*Describe the covariate-relevant population characteristics of the human research participants (e.g. age, genotypic information, past and current diagnosis and treatment categories). If you filled out the behavioural & social sciences study design questions and have nothing to add here, write "See above."*
Recruitment	*Describe how participants were recruited. Outline any potential self-selection bias or other biases that may be present and how these are likely to impact results.*
Ethics oversight	*Identify the organization(s) that approved the study protocol.*

Note that full information on the approval of the study protocol must also be provided in the manuscript.

Field-specific reporting

Please select the one below that is the best fit for your research. If you are not sure, read the appropriate sections before making your selection.

☒ Life sciences ☐ Behavioural & social sciences ☐ Ecological, evolutionary & environmental sciences

For a reference copy of the document with all sections, see nature.com/documents/nr-reporting-summary-flat.pdf

Life sciences study design

All studies must disclose on these points even when the disclosure is negative.

Sample size	No statistical method was used to predetermine sample size. These sample sizes were considered sufficient based on previous experience and assuming the clonal nature of the model organism Hydra and the biological variation behind.
Data exclusions	Only on mouth-opening response of the bud to presenting a single artemia to the parent (Fig. 3B) analyses across bud developmental time-points, the additional control for ensuring that no components from the parent mouth-opening was externally influencing the bud's mouth opening data was not included in the analysis plots. However, it has been mentioned in the methods and is available on datasets as well. It was not presented to simplify the data presentation and due to the fact that it did not change the outcome of the message of the figure. The primary control was the experiment where the parent head was cut and the polyp with the bud presented with GSH.
Replication	All findings presented in the manuscript were replicated as described in Materials and methods and shown in the legends as number of replicates n. All results coming from replication were reproducible and are completely reported here.
Randomization	All the animals employed in the study were kept at the same environmental conditions (temperature, light, feeding regime). All polyps of a

Randomization | given strain are genetically identical (clonal). Moreover, all polyps of a given strain used for a certain experiment came from one population (mass culture), therefore were equally treated before and during the experiment.

Blinding | A fully double-blind design was not implemented, as the study did not involve treatment allocation in the conventional sense but rather developmental time-point comparisons and direct pgysiological measurements. Behavioral outcomes were defined using predefined, binary criteria, minimizing subjective interpretation. However, in case of mouth-opening response time measurements for the N6 ablation data, blinding was carried out to reduce any bias. Calcium imaging and neural activity analyses were based on quantitative signal extraction using consistent analysis pipelines, further reducing potential observer bias. Data analysis was performed using automated or semi-automated pipelines where possible.

Reporting for specific materials, systems and methods

We require information from authors about some types of materials, experimental systems and methods used in many studies. Here, indicate whether each material, system or method listed is relevant to your study. If you are not sure if a list item applies to your research, read the appropriate section before selecting a response.

Materials & experimental systems

n/a	Involved in the study
☐	☒ Antibodies
☒	☐ Eukaryotic cell lines
☒	☐ Palaeontology and archaeology
☐	☒ Animals and other organisms
☒	☐ Clinical data
☒	☐ Dual use research of concern
☒	☐ Plants

Methods

n/a	Involved in the study
☒	☐ ChIP-seq
☒	☐ Flow cytometry
☒	☐ MRI-based neuroimaging

Antibodies

Antibodies used | Commercially available antibodies, as reported in the paper, were used in this study.

[Primary antibodies : rabbit anti-RFamide (#T-4322, Biozol) , rabbit anti-DsRed (#632496, Takara), chicken anti-GFP (#GFP-1010, Aves Labs). Secondary antibodies: Alexa Fluor™ 546 donkey anti-rabbit (#A10040, Invitrogen), Alexa Fluor™ 488 goat anti-rabbit (#A11008, Invitrogen), Alexa Fluor™ 488 goat anti-chicken (#A11039, Invitrogen), Alexa Fluor™ 546 goat anti-chicken (#A11040, Invitrogen)]

Validation | Rabbit anti-DsRed (Takara, Cat# 632496, RRID:AB_10013483) and chicken anti-GFP (Aves Labs, Cat# GFP-1010, RRID:AB_2307313) are validated by the manufacturers for IF/IHC/WB and widely cited (CiteAb). Both recognize heterologous fluorescent proteins (DsRed/mCherry, GFP), and are therefore species-independent and suitable for Hydra transgenic samples.
Rabbit anti-RFamide (Biozol, Cat# T-4322) targets the conserved RFamide neuropeptide motif present in cnidarians. RFamide immunoreactivity is an established neuronal marker in Hydra (e.g., Grimmelikhuijzen, 1985; Takaku, 2017). Specificity is supported by conserved peptide recognition and prior immunostaining studies.

Animals and other research organisms

Policy information about studies involving animals; ARRIVE guidelines recommended for reporting animal research, and Sex and Gender in Research

Laboratory animals | Hydra vulgaris strain AEP, males and females. Transgenic lines of N6 and N4, GCaMP and NTR were used from previously published Giez et al., CurrBiol 2023. Lines from the constructs t12588::GCaMP6s, t12588::mutNTR , and myc3:GFP//act:DsRED were created in the current study, and is described in the paper. All the lines are available upon request to the corresponding authors.

Wild animals | No wild animals were used in this study.

Reporting on sex | Information on sex of animals has not been collected in this study. Under our experimental conditions, animals were maintained in a predominantly asexual state, where sex is not stable or defining biological variable. Our study aimed to characterize general principles of neural circuit formation and behavior, which are not known to exhibit sex-specific differences in Hydra.

Field-collected samples | No field-collected samples were used in this study.

Ethics oversight | No ethical approval was required to perform experiments with the invertebrate Hydra .

Note that full information on the approval of the study protocol must also be provided in the manuscript.

Plants

Seed stocks	*Report on the source of all seed stocks or other plant material used. If applicable, state the seed stock centre and catalogue number. If plant specimens were collected from the field, describe the collection location, date and sampling procedures.*
Novel plant genotypes	*Describe the methods by which all novel plant genotypes were produced. This includes those generated by transgenic approaches, gene editing, chemical/radiation-based mutagenesis and hybridization. For transgenic lines, describe the transformation method, the number of independent lines analyzed and the generation upon which experiments were performed. For gene-edited lines, describe the editor used, the endogenous sequence targeted for editing, the targeting guide RNA sequence (if applicable) and how the editor was applied.*
Authentication	*Describe any authentication procedures for each seed stock used or novel genotype generated. Describe any experiments used to assess the effect of a mutation and, where applicable, how potential secondary effects (e.g. second site T-DNA insertions, mosiacism, off-target gene editing) were examined.*
